# Seasonal Dynamics in Soil Properties Along a Roadway Corridor: A Network Analysis Approach

**DOI:** 10.3390/ma18081708

**Published:** 2025-04-09

**Authors:** Ibrahim Haruna Umar, Ahmad Muhammad, Hang Lin, Jubril Izge Hassan, Rihong Cao

**Affiliations:** 1 School of Resources and Safety Engineering, Central South University, Changsha 410083, China; ibrahimharunaumar@yahoo.com; 2 Department of Civil Engineering, Faculty of Engineering, Aliko Dangote University of Science and Technology, Wudil 713101, Kano State, Nigeria; 3 Department of Civil Engineering, Kano State Polytechnic, Kano 700222, Kano State, Nigeria; ahmad.civilengr@gmail.com; 4 Department of Geology, Faculty of Physical Sciences, Ahmadu Bello University Zaria, Zaria 810211, Kaduna State, Nigeria; jihassan@abu.edu.ng

**Keywords:** soil spatial variability, seasonal variation, roadway, California bearing ratio (CBR), network analysis, centrality measures

## Abstract

Understanding soil properties’ spatial and temporal variability is essential for optimizing road construction and maintenance practices. This study investigates the seasonal variability of soil properties along a 4.8 km roadway in Maiduguri, Nigeria. Using a novel integration of network analysis and geotechnical testing, we analyzed nine soil parameters (e.g., particle size distribution (PSD), Atterberg limits, California bearing ratio) across wet (September 2024) and dry (January 2021) seasons from 25 test stations. Average Atterberg limits (LL: 22.8% wet vs. 17.5% dry; PL: 18.7% wet vs. 14.7% dry; PI: 4.2% wet vs. 2.8% dry; LS: 1.8% wet vs. 2.3% dry), average compaction characteristics (MDD: 1.8 Mg/m^3^ wet vs. 2.1 Mg/m^3^ dry; OMC: 12.3% wet vs. 10% dry), and average CBR (18.9% wet vs. 27.5% dry) were obtained. Network construction employed z-score standardization and similarity metrics, with multi-threshold analysis (*θ* = 0.05, 0.10, 0.15) revealing critical structural differences. During the wet season, soil networks exhibited a 5.0% reduction in edges (321 to 305) and density decline (1.07 to 1.02) as thresholds tightened, contrasting with dry-season networks retaining 99.38% connectivity (324 to 322 edges) and stable density (0.99). Seasonal shifts in soil classification (A-4(1)/ML wet vs. A-2(1)/SM dry) underscored moisture-driven plasticity changes. The findings highlight critical implications for adaptive road design, emphasizing moisture-resistant materials in wet seasons and optimized compaction in dry periods.

## 1. Introduction

Understanding soil properties’ spatial and temporal variability is paramount in various engineering applications, particularly in designing and constructing transportation infrastructure such as roads and highways [1]. Soil characteristics, including particle size distribution, Atterberg limits, compaction behavior, and bearing capacity, can significantly influence the performance and durability of road structures. These properties are not static but can exhibit considerable variations due to environmental factors, most notably seasonal changes in climatic conditions [2]. Seasonal fluctuations in temperature, precipitation, and moisture content can profoundly impact soil behavior, leading to alterations in physical and mechanical properties [3]. During wet seasons, soils may experience higher moisture levels, potentially affecting particle cohesion, plasticity, and compaction characteristics. Conversely, dry seasons can lower moisture content, impacting soil strength, bearing capacity, and erosion resistance [4]. A failure to account for these seasonal variations can lead to suboptimal design decisions, compromised construction quality, and increased maintenance requirements for road infrastructure [5]. Northern Nigeria, characterized by a Sahelian climate, experiences distinct wet and dry seasons, making it an ideal setting for investigating the effects of seasonal variations on soil properties [6]. The region’s semi-arid conditions, influenced by the Harmattan wind and the West African Monsoon system, provide contrasting environmental features that can significantly impact soil behavior [7].

Network analysis techniques have become integral to civil engineering, facilitating the optimization of infrastructure design, project management, and sustainability assessments [8]. This literature review explores the evolution and application of network analysis methods within the field, highlighting their significance and recent advancements. In project management, methods like the critical path method (CPM) and program evaluation and review technique (PERT) have become essential tools for planning and controlling construction activities, utilizing network diagrams to represent task interdependencies and facilitate resource allocation [9]. Luleci et al. investigate the integration of artificial intelligence (AI), particularly generative adversarial networks (GANs), which have revolutionized structural health monitoring (SHM) by generating synthetic data for model training, thereby improving the accuracy of damage detection and structural assessment [10]. The investigation by Hewa Welege et al. on social network analysis (SNA) has proven valuable in mapping stakeholder relationships within sustainable construction projects, optimizing collaboration strategies, and enhancing project outcomes [11]. Dandy et al. investigate the water distribution systems, and network analysis techniques incorporating linear and nonlinear programming, along with genetic algorithms, which have enabled the design of cost-effective and efficient pipe networks [12]. Zhou et al. examine spatial analysis methods, such as space syntax, which have become instrumental in urban planning by evaluating street network connectivity and spatial configurations to improve accessibility and functionality [13]. These diverse network analysis applications have collectively enhanced civil engineering practices’ efficiency, sustainability, and innovation, demonstrating the technique’s versatility and significance in addressing complex infrastructure challenges.

A longitudinal soil sampling study was conducted in Maiduguri, Nigeria to examine seasonal dynamics of soil properties along a 4.8 km roadway near the Alau Dam. The research aimed to investigate the contrasting effects of dry and wet seasons on soil moisture, chemical composition, and physical structure, addressing a critical gap in understanding how seasonal variations impact road construction corridors [14,15,16]. For instance, studies conducted in Virginia, North Dakota, New York, and Vermont have reported increased bulk density and reduced water infiltration at road-edge positions compared to non-road edges, attributed to construction, traffic, and vibrations consolidating soils. These changes potentially compromise vegetation establishment and road infrastructure integrity [17]. While existing research has acknowledged seasonal influences on soil properties, comprehensive investigations specific to road construction corridors remain limited. Current network analysis techniques have primarily been applied to agricultural and environmental contexts, overlooking the unique challenges presented by transportation infrastructure projects [18]. The study recognizes that seasonal variations in soil moisture, density, and compaction characteristics demand careful consideration during road design and construction. By providing insights into these dynamic soil properties, the research seeks to inform more robust soil management strategies and enhance the long-term durability of transportation infrastructure in regions with pronounced seasonal environmental shifts.

**Figure 1:**
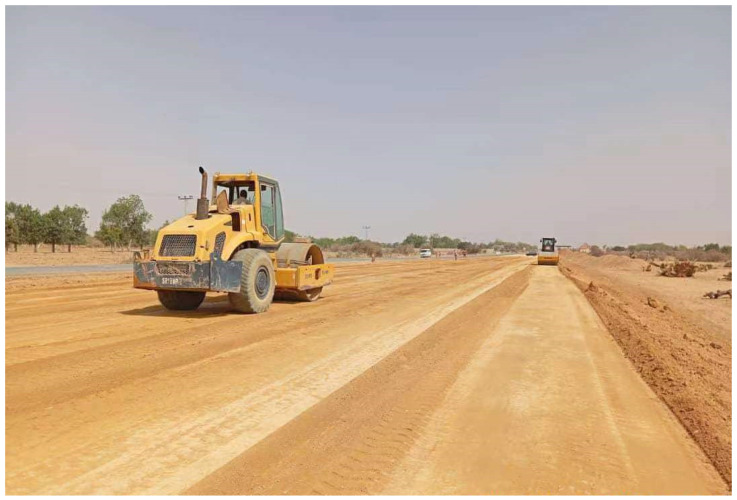
Workers compacting soil at a roadway.

The novelty of the presented study lies in its comprehensive approach to investigating the effects of seasonal variations on soil properties along a specific road construction site in northern Nigeria, utilizing both traditional soil analysis methods and advanced network analysis techniques. By analyzing soil samples collected during different seasons and employing network analysis to explore the spatial variability and interconnectedness of soil characteristics among test stations, the study offers new insights into the multidimensional nature of soil spatial variability along transport corridors. The application of network analysis, including centrality measures and community detection algorithms, to study the structure and properties of the network of test stations based on soil characteristics is a novel contribution to the field. This approach allows for a more nuanced understanding of the interconnectedness and similarity among test stations. This can inform more effective road design, construction practices, and soil management strategies that account for soil properties’ complex spatial and temporal variability. Figure 1 shows the soil compaction process during roadway construction, demonstrating the critical geotechnical engineering technique influencing soil strength, permeability, and settlement behavior. The workers use standard compaction equipment to achieve optimal soil density, a crucial step in preparing the roadway’s foundation.

While prior studies have examined soil spatial variability in agricultural or environmental engineering, limited attention has been devoted to transportation infrastructure corridors, where seasonal hydrological dynamics directly impact geotechnical performance. Specifically, existing literature lacks a systematic integration of network analysis with traditional geotechnical parameters (e.g., Atterberg limits, California bearing ratio) to quantify seasonal soil property interdependencies along roadways. This gap is particularly pronounced in semi-arid regions like northeastern Nigeria, where extreme seasonal shifts—exacerbated by events such as dam failures—create unique challenges for infrastructure resilience. Prior work has yet to address how moisture-driven plasticity changes and particle redistribution influence networked soil behavior across wet and dry seasons, limiting the development of adaptive design strategies for vulnerable transport networks. The study was designed to address this gap through three interconnected objectives: 1. To characterize seasonal variations in soil properties (particle size distribution, Atterberg limits, compaction characteristics, and CBR) along a flood-affected roadway in Maiduguri, Nigeria; 2. To apply network analysis—employing centrality measures and multivariate thresholds—to identify spatial and temporal patterns in soil property interdependencies; 3. To evaluate the engineering implications of seasonal soil dynamics for road design, including compaction optimization. These objectives collectively advance the application of network science in geotechnical engineering while addressing region-specific challenges posed by seasonal extremes.

## 2. Background of Study

The study area, strategically positioned near Maiduguri, encompasses a critical transitional zone where river networks intersect anthropogenic infrastructure, presenting an ideal locale for examining soil property dynamics. Extending toward the Alau Dam, the corridor offers a comprehensive perspective on soil transformation mechanisms influenced by hydrological and climatic variations. The region’s geotechnical landscape was profoundly altered by the catastrophic Alau Dam collapse on the 10 September 2024, which precipitated extensive flooding and induced significant geomorphological disruptions. This event underscored the complex interactions between hydrological systems and soil properties, highlighting the vulnerability of infrastructural and environmental systems [19]. Situated within the semi-arid Sahelian belt, Maiduguri experiences pronounced seasonal variations, with an average annual precipitation of 452 mm predominantly concentrated in the wet season (June–September). The local soil composition—characterized by sandy loams with minimal organic content and variable clay fractions—renders the landscape intrinsically susceptible to moisture-induced transformations [20]. The research site’s geological and hydrological attributes provide a nuanced framework for investigating soil property evolution, offering critical insights into environmental management and sustainable land-use strategies in climatically dynamic regions.

**Figure 2:**
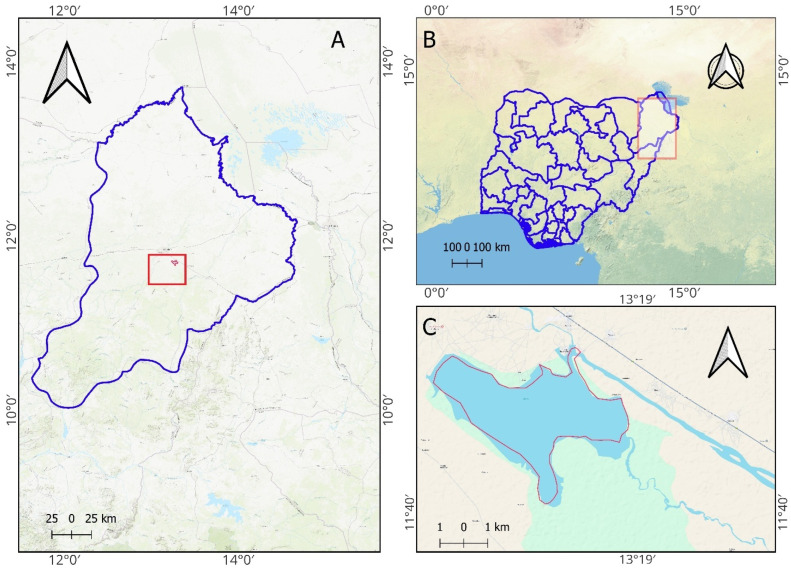
Study area showing the geographical location along the river network in Maiduguri, Borno State, Nigeria. (**A**) a map showing a region with two labelled locations: Maiduguri City and Alau Dam, a red square locating the dam indicates a path or line of sight between them. (**B**) the map of Nigeria and Maiduguri City, a red square located in Maiduguri state, Nigeria. (**C**) provides a geographical feature for the location of Alau Dam within Maiduguri and Nigeria.

The Alau Dam, constructed between 1984 and 1986, is a critical water source for irrigation, domestic use, and fisheries in Maiduguri and its environs. The Alau Dam, a vital water reservoir fed by the Konduga River and tributaries from Niger and Chad, historically mitigated seasonal water scarcity. However, its collapse released torrents of water that inundated vast areas, saturating soils and redistributing sediments across Maiduguri’s urban and peri-urban landscapes [21]. Figure 2A shows a map showing a region with two labelled locations: Maiduguri City and Alau Dam, a red square locating the dam indicates a path or line of sight between them. Figure 2B shows the map of Nigeria and Maiduguri City, a red square located in Maiduguri state, Nigeria. Figure 2C provides a geographical feature for the location of Alau Dam within Maiduguri and Nigeria, helping to understand its position and scale relative to the surrounding areas.

**Figure 3:**
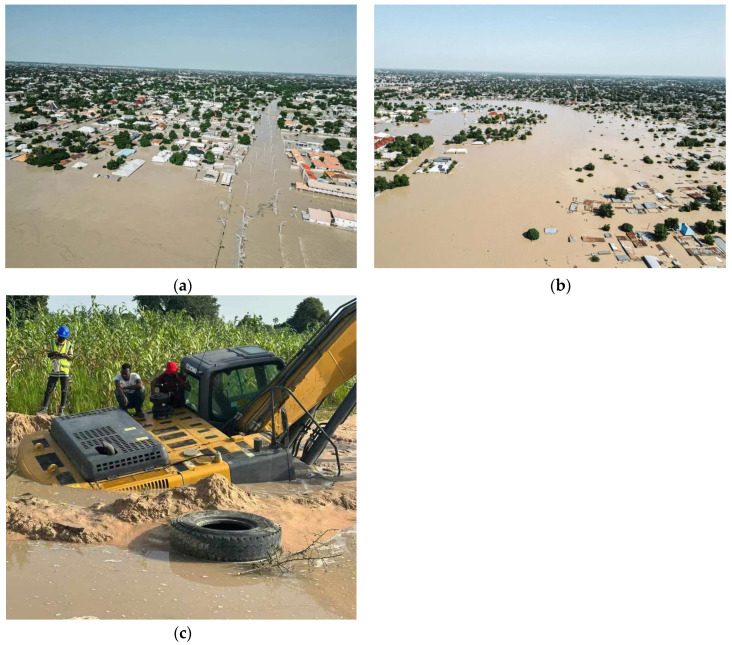
Views of severe flooding in Maiduguri City, Nigeria: (**a**) flooded area, (**b**) structures submerged, (**c**) heavy machinery stuck in mud. Source: National Emergency Management Agency (NEMA).

The flooding underscored the interplay between climate extremes and soil behavior. Pre-existing vulnerabilities—such as inadequate drainage systems and deforestation—amplified the dam’s collapse effects, destabilizing soils and compromising road foundations [22]. Network analysis revealed moderate interconnectivity in soil properties across stations, suggesting that flood-induced homogenization was spatially limited. This spatial variability necessitates site-specific engineering solutions, such as reinforced embankments in erosion-prone zones and moisture-resistant materials in urban areas. Figure 3a–c powerfully captures the severity of the flooding and its impact on the community, infrastructure, and environment of Maiduguri City, Nigeria.

### 2.1. Study Area and Site Description

The study implemented a systematic sampling approach along a 4.8 km roadway in Maiduguri, strategically establishing 25 test stations to capture spatial soil property variations. The sampling design reflected the complex environmental gradient from the urban core to the Alau Dam reservoir, accommodating urban clustering, river meandering, and hydrological influences. In the urban core (stations 1–10), denser station placement captured flood-induced soil heterogeneity characteristics of densely populated areas with significant anthropogenic modifications. The rural–urban transition zone (stations 11–20), is strategically aligned with river meanders to monitor sediment deposition and geotechnical property variations. Stations near the Alau Dam (21–25) maintained consistent spacing, accounting for coarser sediments and reduced human interference. This variable sampling strategy emerged from preliminary hydrogeological reconnaissance, targeting flood stagnation patterns and sediment sorting processes. The methodology’s nuanced spatial approach enhances research transparency and reproducibility, providing a comprehensive framework for investigating soil property dynamics across a complex environmental gradient. By integrating variable station densities and strategic positioning, the study captures the intricate interactions between hydrological processes, urban development, and soil characteristics in a dynamic landscape.

### 2.2. Field Sampling and Laboratory Testing

**Figure 4:**
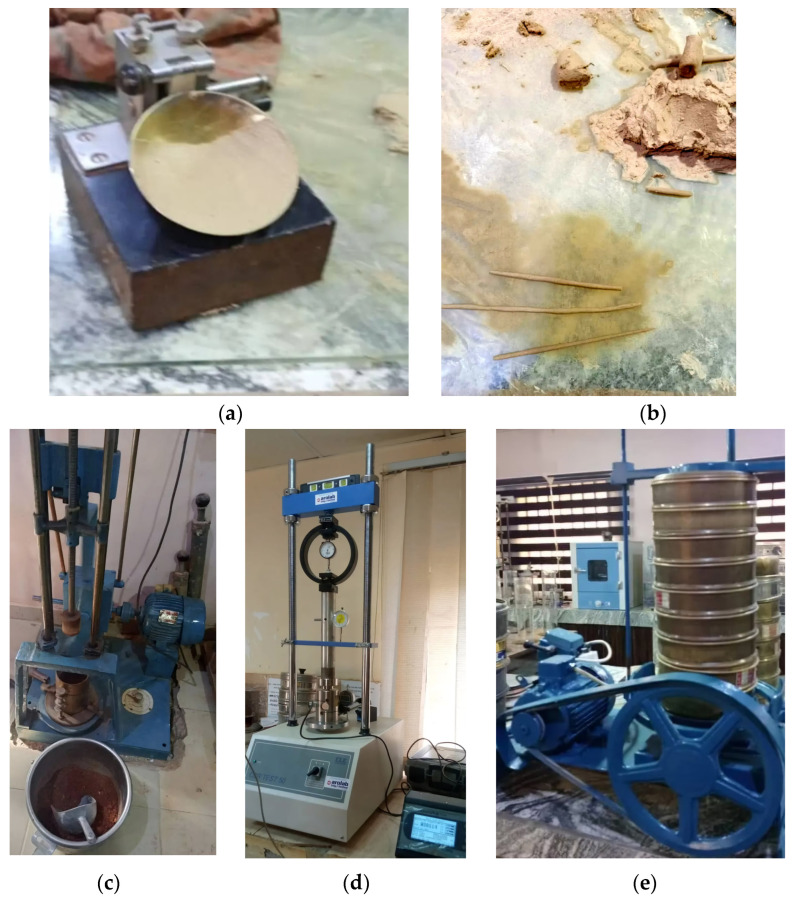
Test equipment and procedures: (**a**) Casagrande apparatus during the liquid limit test, (**b**) plastic limit experimental samples, (**c**) compaction mold preparation, (**d**) CBR testing apparatus, (**e**) sieve analysis setup. All tests followed ASTM standards (Table 1).

The study conducted soil sampling during two climatically distinct periods: the dry season on 14 January 2021, and the wet season on 24 September 2024. The dry season sampling, characterized by Harmattan winds, minimal precipitation, and low humidity (around 25%), captured soil conditions under high temperature variability and reduced leaching potential. The subsequent wet season sampling, influenced by the West African Monsoon, presented markedly different environmental conditions. Elevated temperatures, substantial daily precipitation (approximately 175 mm), and increased humidity (around 70%) defined this period. The southwesterly monsoon winds further distinguished the seasonal attribute. By maintaining consistent sampling locations across these contrasting seasons, the research design facilitated direct temporal comparisons. The methodology was strategically conceived to elucidate seasonal variations in soil properties, including moisture content, organic matter decomposition, nutrient mobility, and potential redox condition transformations. The comprehensive sampling approach, supported by standardized test equipment and protocols, as illustrated in Figure 4 and detailed in Table 1, provides a robust framework for investigating the dynamic geomorphological characteristics of Maiduguri’s urban and peri-urban landscapes.

### 2.3. Network Analysis Method Using Python (Version 3.9.7, Python Software Foundation, Wilmington, DE, USA)

#### 2.3.1. Network Construction and Data Preprocessing

First, all soil properties were standardized using the z-score standardization calculated using Equation (1). This standardization step is crucial because the soil properties measured (Atterberg limits, specific gravity, OMC, MDD, CBR, etc.) have different units and ranges of values. Without standardization, properties with larger numerical values would dominate the similarity calculations. The z-score standardization transforms all properties to have a mean of 0 and a standard deviation of 1 as a reference to Elmore and Richman [28], ensuring they contribute equally to the similarity measure.

(1) $${x^{\prime }}_{ik}=\frac{x_{ik}-\mu_{k}}{\sigma_{k}}$$

where

$x_{ik}^{’}$ = standardized value of the *k*th soil property at station *i*
$x_{ik}$ = original value
$\mu_{k}$ = mean of the *k*th soil property across all stations
$\sigma_{k}$ = standard deviation of the *k*th soil property across all stations

The network analysis in this study employed similarity-based networks, with the 25 test stations serving as nodes and edges weighted by the similarity of soil properties between stations. The analysis utilized two key centrality measures—betweenness and closeness centrality—to understand the spatial relationships and importance of different stations in both wet and dry seasons.

#### 2.3.2. Network Construction and Similarity Measures

The soil property analysis employed a sophisticated network representation defined as *G = (V, E, W)*, conceptualizing the 25 test stations as a complex geotechnical system. This mathematical framework transcended traditional linear sampling limitations through a multi-scale investigative approach. The methodology integrated targeted geotechnical investigations, geostatistical modeling, and advanced computational techniques. Kriging interpolation and 3D numerical simulations addressed spatial variability, accounting for anisotropic soil property variations. Supplementary sampling at critical geological transition zones enhanced the analytical precision [29]. Network analysis revealed nuanced station clusters, demonstrating sensitivity to environmental and anthropogenic modifications. Specifically, stations near stabilized zones exhibited distinct behavioral communities during wet-season conditions, validating the method’s sophisticated analytical capacity. Future implementation includes real-time in situ sensor monitoring to track moisture and deformation dynamics, enabling adaptive model refinement. Coupled hydro-mechanical simulations will facilitate a comprehensive evaluation of long-term environmental impacts, ensuring rigorous adherence to sustainability protocols. This layered, interdisciplinary approach synthesizes geotechnical engineering principles with advanced computational methodologies, providing a robust framework for understanding complex soil property interactions across dynamic environmental gradients.

The network model conceptualizes each vertex *vi* as a spatial referent encapsulating comprehensive soil property measurements, including Atterberg limits, specific gravity, optimum moisture content, maximum dry density, and CBR values. These vertices constitute the fundamental analytical units, representing discrete test station locations along the roadway. The edge set *E* delineates interconnections between test stations, with each edge *eij* representing a relationship derived from comparative soil property characteristics. The accompanying weight set *W* introduces a sophisticated dimensionality by quantifying inter-station similarity through normalized weights within the [0,1] range. This weighted network architecture enables multifaceted analytical approaches, facilitating nuanced investigations of spatial soil property relationships. By transforming raw geotechnical data into a dynamic graph representation, the methodology permits the comprehensive examination of seasonal variations, centrality assessments, and network responses to differential similarity thresholds. The approach transcends traditional linear sampling methodologies, providing a robust computational framework for understanding complex geotechnical spatial interactions across varying environmental contexts.

Let *G = (V, E, W)* represent the network

where:

*V* = set of vertices (25 test stations)
*E* = set of edges connecting stations
*W* = set of weights representing the similarity between stations

Network analysis using Euclidean distance is widely employed for studying the relationships and similarities between different entities or data points based on their attributes or characteristics [30,31]. This method can be applied to investigate the spatial variability and interconnectedness of soil characteristics among the test stations. The Euclidean distance is a metric that measures the straight-line distance between two points in a multidimensional space, where each dimension represents a different soil property or variable. This distance is calculated using the Pythagorean theorem, considering the differences in the values of each soil property between the two stations. The similarity weight $w_{ij}$ between stations *i* and *j* can be calculated using the Euclidean distance between their standardized soil properties, which is calculated using Equation (2) as the theoretical basis for similarity-based networks. This is now supported by Newman and Papadimitriou et al. [32,33].

(2) $$w_{ij}=1-\frac{\sqrt{\sum_{k=1}^{n}{\left(x_{ik}-x_{jk}\right)}^{2}}}{\max_{i,j}\sqrt{\sum_{k=1}^{n}{\left(x_{ik}-x_{jk}\right)}^{2}}}$$

where

$x_{ik}$ = *k*th standardized soil property at station *i*
*n* = number of soil properties considered

The denominator normalizes the weights to [0,1].

Essentially, subtract the value of each property from one point to another, square these differences, sum them up, and then take the square root. This is a way to turn distance into similarity. The closer the two stations (smaller distance), the higher their similarity. For example, the less you travel, the more similar two things are.

#### 2.3.3. Betweenness Centrality (BC)

Betweenness centrality (BC) is a widely used measure in network analysis that quantifies the importance of a node (in this case, a soil test station) based on its role as a bridge or connector within the network [34]. It is handy for identifying critical nodes that facilitate the flow of information or resources through the network [35]. Betweenness centrality can provide valuable insights into the importance of specific test stations and their potential to influence or mediate the relationships between other stations within the network [36]. Betweenness centrality is calculated by considering the shortest paths between all pairs of nodes in the network [37]. For a given node, its betweenness centrality is proportional to the shortest paths that pass through it. Nodes with higher betweenness centrality values act as bridges or gateways, connecting different network parts that would otherwise be disconnected or have longer paths. For a node *v*, betweenness centrality is calculated as given by Equation (3), as per Papadimitriou et al. [33].

(3) $$BC(v)=\sum_{s\neq v\neq t}\frac{\sigma_{st}(v)}{\sigma_{st}}$$

where

$\sigma_{st}$ = total number of shortest paths between nodes *s* and *t*
$\sigma_{st}(v)$ = number of shortest paths passing through node *v*

#### 2.3.4. Closeness Centrality (CC)

Closeness centrality (CC) is another important measure in network analysis that quantifies the centrality or accessibility of a node (in this case, a soil test station) within the network [38]. It is based on the concept of average distance or closeness, which measures how close a node is to all other nodes in the network [39]. Closeness centrality can provide insights into the relative importance or influence of specific test stations based on their proximity or ease of access to other stations within the network [40]. The closeness centrality of a node is calculated as the inverse of the sum of the shortest path distances between that node and all other nodes in the network. Nodes with higher closeness centrality values are considered more central or accessible, as they have shorter average distances to other nodes in the network. For a node *v*, closeness centrality is calculated using Equation (4), as used by Liu et al. and Lafhel et al. [41].

(4) $$CC(v)=\frac{n-1}{\sum_{u\neq v}d(v,u)}$$

where:

*n* = total number of nodes
*d(v,u)* = shortest path distance between nodes *v* and *u*

The sum represents the total distance from *v* to all other nodes

For weighted networks, the distance *d(v,u)* is calculated using the inverse of similarity weights:

$$d(v,u)=\frac{1}{w_{vu}}$$

#### 2.3.5. Threshold Analysis

The threshold analysis in our study provides a systematic approach to understanding the network structure at different levels of soil property similarity. By applying different similarity thresholds (*θ* = 0.05, 0.10, and 0.15), we can examine how the network’s connectivity patterns evolve and identify robust relationships between test stations. The study examined network structure at different similarity thresholds (*θ*). An edge exists between stations *i* and *j* if

$$e_{ij}=\left\{\begin{array}{ll} 1 & \mathrm{if}\;w_{ij}\geq \theta \\ 0 & \mathrm{otherwise} \end{array}\right.$$

The analysis reveals distinct patterns across different thresholds. When examining the network at *θ* = 0.05, the lower threshold results in high connectivity, with most stations maintaining connections with numerous others. This provides a broad view of soil property relationships, though it may include weaker or less significant relationships in the analysis. The network shows moderate connectivity at the medium threshold of *θ* = 0.10, revealing more meaningful relationships between stations. This threshold effectively filters out weaker connections while maintaining significant patterns, providing a balanced view of soil property similarities across the study area. The network becomes sparser when the threshold increases to *θ* = 0.15, retaining only the most substantial relationships between stations. This higher threshold highlights stations with highly similar soil properties and is particularly useful for identifying core relationships in the network structure.

The network density (D) at each threshold quantifies these changes and is calculated using Equation (5), as given by Liu et al. and Lafhel et al. [41].

(5) $$D=\frac{2|E|}{\left|V|(|V|-1\right)}$$

where

*|E|* = number of edges
*|V|* = number of vertices (25 stations)

Threshold analysis provides a sophisticated methodological approach for exploring soil property relationships across varying similarity levels. By systematically examining network structures at different thresholds, the approach unveils the hierarchical complexity of spatial soil property interactions. The multi-threshold methodology enables a nuanced detection of consistently similar stations, facilitating comprehensive comparisons between wet and dry seasonal networks. This approach transcends traditional single-threshold analyses, revealing the dynamic nature of soil property relationships across spatial and temporal dimensions. The mathematical framework not only quantifies seasonal variations in network connectivity but also identifies critical stations based on their structural positioning. By bridging theoretical network analysis principles with practical geotechnical applications, the methodology offers unprecedented insights into soil property dynamics. The comprehensive analysis demonstrates how seasonal changes fundamentally transform soil property relationships, providing critical information for infrastructure monitoring, maintenance strategies, and understanding landscape-scale geomorphological processes.

#### 2.3.6. Analytical Process

The network analysis methodology employs a systematic approach to investigating soil property relationships through Euclidean distance computational techniques. Data organization involves constructing a comprehensive matrix representing test stations and their corresponding soil property measurements, necessitating preliminary standardization to ensure equitable property contributions.

The analytical process involves generating a distance matrix capturing pairwise station dissimilarities, subsequently constructing a network wherein nodes represent test stations and edges reflect inter-station connections based on predefined similarity thresholds. This computational framework enables sophisticated spatial relationship investigations. Advanced analytical techniques are applied to network structures, including centrality measures for identifying influential stations, community detection algorithms for clustering stations with analogous soil properties, and path analysis for examining connectivity dynamics. The similarity threshold selection critically influences network interpretation, with lower thresholds producing more interconnected networks and higher thresholds revealing the most substantial property relationships. The methodology’s nuanced approach transforms raw geotechnical data into a mathematically rigorous framework, facilitating the comprehensive understanding of spatial soil property interactions across varied environmental settings.

#### 2.3.7. Structure of the Network

**Figure 5:**
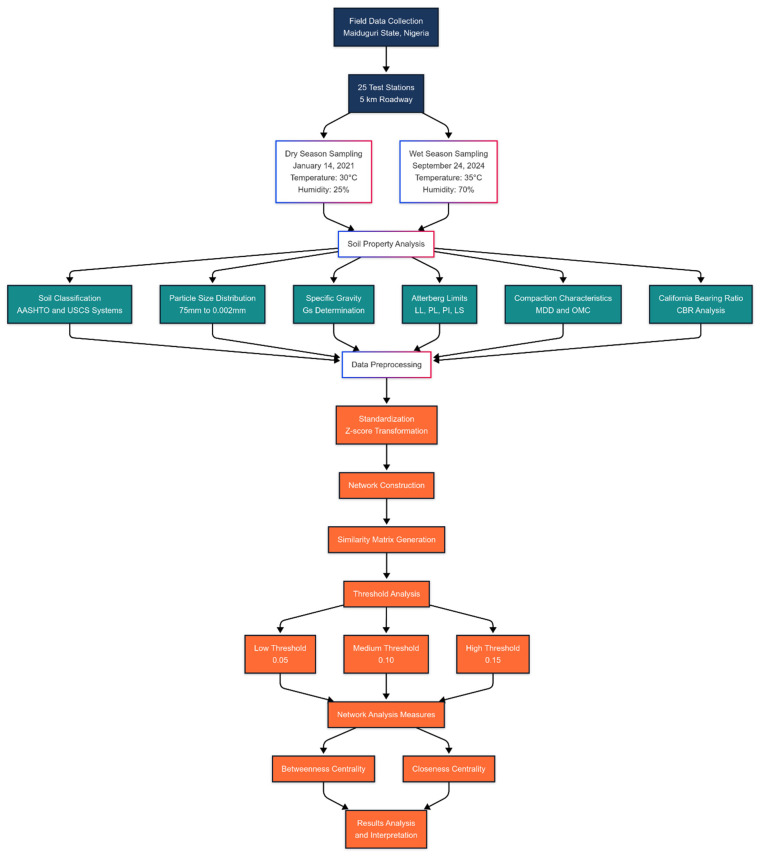
A flowchart of the methodology of this network analysis study of soil properties.

The network representation transforms test station data into a graph-based analytical framework, where nodes signify individual stations and edges illustrate property similarities. By normalizing soil properties and employing Euclidean distance metrics, the methodology enables the precise quantification of inter-station resemblances. The network’s configuration is dynamically determined by computational similarity measures, transcending the original data sequence. Stations exhibiting high property correspondence are interconnected, revealing intricate spatial relationships independent of their initial arrangement. This approach prioritizes inherent geotechnical characteristics over arbitrary positioning. Specialized visualization techniques strategically position nodes and edges, facilitating pattern identification and cluster recognition. The network construction process integrates traditional geotechnical testing methodologies with advanced computational network analysis, providing a sophisticated lens for examining soil property interactions. Figure 5 shows the flowchart, which illustrates the systematic approach taken in the study, from initial field sampling through soil property analysis, network construction, and final analysis. It shows how the process integrates traditional geotechnical testing with modern network analysis techniques.

The network analysis was implemented using Python’s computational ecosystem, with NetworkX (version 2.8.4) serving as the primary library for network construction and analysis. The methodology integrated NumPy (version 1.22.3), Pandas (version 1.4.2), and scikit-learn (version 1.0.2) for data preprocessing, similarity matrix calculations, and advanced computational techniques. Matplotlib (version 3.5.1) and NetworkX’s visualization utilities enabled the comprehensive graphical representation of network structures and centrality measures. The cosine similarity metric, implemented through scikit-learn’s pairwise_distances function, provided robust quantification of multidimensional soil property relationships. This computational approach aligns with contemporary scientific research protocols, emphasizing transparency and computational rigor in geotechnical network analysis.

## 3. Soil Engineering Properties

### 3.1. Soil Classification for Wet and Dry Seasons

The soil classification results along a 4.8 km road alignment, based on the AASHTO (American Association of State Highway and Transportation Officials) and USCS (Unified Soil Classification System) classification systems, are presented. Table 2 provides the soil classification for wet and dry conditions at various test stations (TS) spaced at 200 m intervals along the road alignment. According to Table 2, the predominant soil type along the entire road alignment is classified as A-4(1) or ML (silt) under wet conditions and A-2(1) or SM (silty sand) under dry conditions, based on the AASHTO and USCS systems, respectively. This observation is consistent across all 25 test stations, indicating high uniformity in the soil characteristics along the road alignment. The observed differences in soil classification between wet and dry conditions can be attributed to the influence of moisture content on soil behavior. Soils with higher moisture content tend to exhibit more remarkable plasticity and cohesion, leading to classification as silt (ML) or low-plasticity silt (CL-ML) under the USCS system and as A-4(1) soil under the AASHTO system. Conversely, the reduced moisture content decreases plasticity and cohesion when the soil is dry. This leads to a classification as silty sand (SM) under the USCS system and as an A-2(1) soil under the AASHTO system.

The phenomenon is well documented in the literature and is a common observation in areas with seasonal precipitation and variations in moisture conditions. For example, a study by Muhammad et al. on soil classification in tropical regions reported similar trends, where soils classified as silts or clays during the wet season exhibited characteristics of sandy soils during the dry season [42,43]. The observed changes in soil classification can significantly affect engineering design and construction activities. During the wet season, the predominance of fine-grained soils (ML or A-4(1)) may necessitate additional considerations for drainage, stability, and compaction requirements, as these soils are more susceptible to moisture-related volume changes and potential strength reductions. Conversely, during the dry season, coarser-grained soils (SM or A-2(1)) may require different approaches to compaction and stabilization techniques.

### 3.2. Particle Size Distribution (PSD) for Wet and Dry Seasons

**Figure 6:**
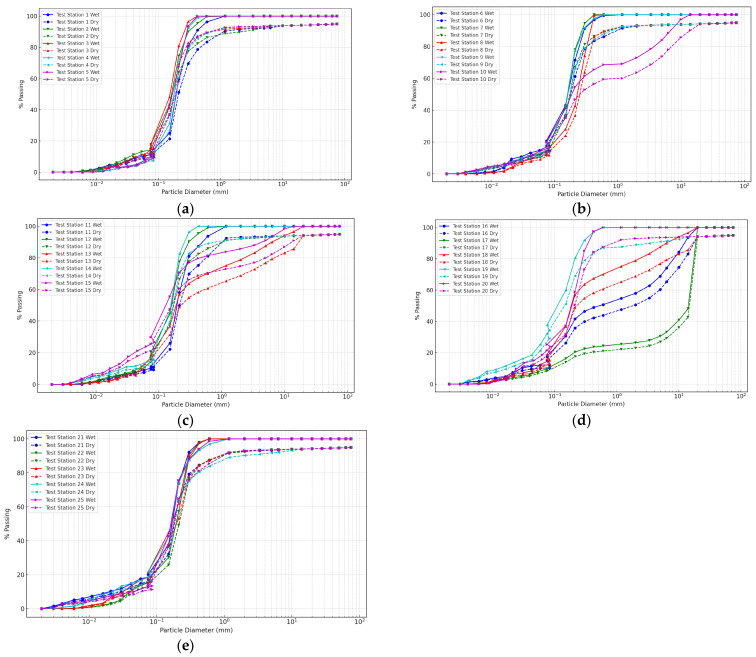
Particle size distribution curve during wet and dry seasons. (**a**). At test stations 1 to 5 (group 1). (**b**). At test stations 6 to 10 (group 2) (**c**). At test stations 11 to 15 (group 3). (**d**). At test stations 16 to 20 (group 4). (**e**). At test stations 21 to 25 (group 5).

PSD is a critical parameter in soil characterization since it affects various properties, including permeability, shear strength, compaction, and bearing capacity. These are essential considerations in road construction projects. Figure 6a–e cover a wide range of particle sizes, from 75 mm to 0.002 mm, encompassing coarse gravel, sand, silt, and clay fractions. The data present quantitative variables as percentage passing values for each particle size at every test station. These values indicate the cumulative percentage of soil particles finer than the corresponding particle size. For example, during the wet season at test station 1, all soil particles were finer than 75 mm, while only 2.598167% were finer than 0.011 mm. Also, PSD curves are crucial for comprehending soil behavior in diverse environmental conditions, including wet and dry seasons. These curves demonstrate the proportion of different particle sizes within a soil sample, significantly affecting soil properties such as moisture movement, contaminant transport, and erosion. Seasonal variations, especially between wet and dry seasons, can substantially impact the PSD of soils, resulting in alterations to soil physical properties.

From Figure 6a–e, it becomes apparent that the magnitude and direction of seasonal variation differ across particle sizes and test stations. Certain test stations display more significant differences between wet and dry seasons, while others exhibit minor variations. For instance, at test station 1, the percentage passing for the particle size of 0.6 mm differed by 13.88424% (96.33212% in the wet season; 83.44788% in the dry season). This finding suggests a higher percentage of finer particles during the wet season than the dry season for this specific particle size and test station.

Seasonal variations significantly influence soil particle size distribution, with the wet season characterized by a higher proportion of finer particles (silt and clay fractions) compared to the dry season. For example, at test station 1, the rate of particles passing through the 0.075 mm sieve (silt fraction) is 12.6684% during the wet season, compared to 10.7916% during the dry season. This phenomenon is attributed to multiple mechanisms, including water-induced particle dispersion, selective particle transport through precipitation, and the swelling of expansive soils [44,45]. The increased presence of finer particles during the wet season demonstrates notable geotechnical implications. Particle size modifications enhance water retention capacities, with finer particles exhibiting higher surface area-to-volume ratios that facilitate increased moisture adsorption and capillary forces. Comparative analysis across test stations consistently revealed higher percentages of particles passing through 0.075 mm sieves during wet season conditions. Moreover, the enhanced concentration of finer particles during the wet season presents significant environmental challenges. The increased specific surface area of smaller particles facilitates contaminant transport, potentially compromising groundwater quality and increasing soil vulnerability to leaching processes [46,47]. Simultaneously, the higher proportion of fine particles escalates soil erosion susceptibility, as these particles are more readily detached and transported by water erosion mechanisms [48,49]. These observations underscore the complex interactions between seasonal hydroclimatic conditions and soil geomorphological characteristics, highlighting the critical importance of understanding particle size distribution dynamics for comprehensive environmental and geotechnical management strategies [50,51].

The dry season demonstrates a pronounced prevalence of coarser particles (sand and gravel fractions) compared to the wet season, characterized by distinct geomorphological transformations. Comparative analysis across test stations reveals significant variations in particle size distributions, with coarser particle concentrations demonstrating notable seasonal fluctuations. For instance, at test station 5, the percentage passing for the 0.6 mm particle size (coarse sand fraction) is 89.51547% during the dry season, while it is 100% during the wet season, indicating a higher concentration of coarser particles in the dry season. The predominance of coarser particles during the dry season stems from multiple geotechnical mechanisms. Reduced moisture content promotes soil compaction, facilitating the aggregation of finer particles into larger clusters and potentially inducing particle breakdown. Diminished lubrication effects during dry conditions contribute to the mechanical restructuring of soil particle configurations [52]. Reduced erosion rates during the dry season enable the accumulation and deposition of coarser particles transported from adjacent geological sources. These observations align with established research documenting the intricate relationship between hydroclimatic conditions and soil particle dynamics [53]. The study’s comprehensive spatial and temporal analysis of particle size distribution across multiple test stations distinguishes it from conventional soil characterization research. By providing site-specific, high-resolution data from a road construction project, the research offers critical insights into seasonal soil property variations, essential for developing robust infrastructure design strategies, soil stabilization techniques, and adaptive construction methodologies.

### 3.3. Specific Gravity for Wet and Dry Seasons

The specific gravity (G_s_) of soil particles is a fundamental property that remains constant regardless of seasonal changes in PSD. The principles of soil mechanics and existing literature on soil properties support this observation. Specific gravity is defined as the ratio of the density of a given substance to the density of water at a specified temperature, typically 4 °C (39.2 °F). The specific gravity of soil particles is an intrinsic property determined by their mineralogical composition and internal structure. It represents the ratio of the particle density to the density of water and is independent of particle size or overall particle size distribution. The specific gravity is a measure of the density of the solid particles. It is not influenced by particle size or relative proportions of different particle size fractions within the soil [54]. The observed seasonal variations in the PSD section are primarily caused by moisture content, aggregation/dispersion processes, erosion, and particle rearrangement. These factors can change the relative proportions of different particle size fractions (such as gravel, sand, silt, and clay) within the soil. Still, they do not affect the fundamental properties of the individual soil particles, including their specific gravity.

**Figure 7:**
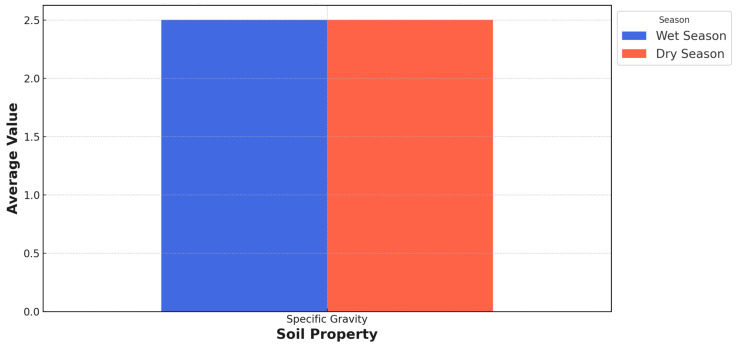
Specific gravity of the soil during wet and dry seasons across all the test stations.

Figure 7 displays the average specific gravity of soil from the 25 test stations during wet and dry seasons as 2.5. Typical specific gravity values for mineral soils composed of quartz and common silicate minerals range from around 2.6 to 2.8 [55]. However, soils with higher organic matter content or the presence of certain minerals, such as iron oxides or heavy metal compounds, may exhibit higher specific gravity values.

This principle is well-established in the literature on soil mechanics and geotechnical engineering. The specific gravity of soil particles is typically considered a constant value for a given soil type, and it is used in various calculations and analyses related to soil behavior, such as density, void ratio, and soil classification [45]. The seasonal variations in PSD can affect other soil properties, such as permeability, shear strength, and compaction characteristics. However, the specific gravity remains unaffected because it is a function of the soil particles’ intrinsic properties rather than their arrangement or particle size distribution within the soil matrix. It is important to note that the specific gravity may vary among different soil types due to their mineralogical compositions and the presence of organic matter or other constituents. However, for a given soil type, the specific gravity remains constant regardless of seasonal changes in PSD or other environmental factors that may affect the particle size distribution.

### 3.4. Atterberg Limits for Wet and Dry Seasons

The Atterberg limits define the consistency of fine-grained soils at different moisture contents. Liquid limit (LL) ranges from 18.1 to 24.3% in the wet season, decreasing to 12.6–18.0% in the dry season as soils become less plastic. The plastic limit (PL) ranges from 13.6 to 19.2% in the wet season, reducing to 8.9–18.0% in the dry season. The plasticity index (PI) shows values of 4.5–8.3% in the wet season, decreasing to 2.1–5.6% in the dry season, indicating lower moisture sensitivity. Linear shrinkage (LS) ranges from 1.1 to 3.9% in the wet season and from 1.5 to 3.1% in the dry season, with drier conditions generally causing more soil shrinkage (Table 3).

**Figure 8:**
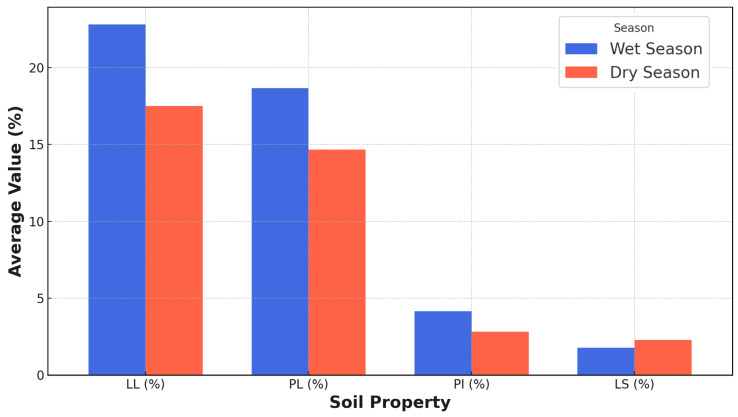
Average Atterberg limits during wet and dry seasons across all the test stations.

The liquid limit (LL) analysis in Figure 8 reveals significant seasonal variations, with average values of 22.8% during the wet season and 17.5% during the dry season. This parameter provides critical insights into soil behavior and engineering properties for road construction applications. The wet season LL value corresponds to inorganic clays of low to medium plasticity (CL) or silts of low plasticity (ML) within the unified soil classification system. Conversely, the dry season LL value aligns more closely with inorganic silts of low plasticity (ML) or non-plastic silty soils (ML-SM). Seasonal fluctuations in liquid limit values reflect complex soil–water interactions and environmental dynamics [56]. Increased moisture content during wet seasons weakens inter-particle forces, facilitating greater particle rearrangement and enhancing soil plasticity [57]. Dry season conditions promote particle compaction, reducing moisture retention and constraining the soil’s ability to transition to a liquid state [54].

The plastic limit (PL) analysis in Figure 8 reveals significant seasonal variations, with average values of 18.7% during the wet season and 14.7% during the dry season. This parameter provides critical insights into soil behavior and engineering properties for road construction applications. The wet season PL value corresponds to inorganic clays of low to medium plasticity (CL) or silts of medium plasticity (ML) within the unified soil classification system. Conversely, the dry season PL value aligns more closely with inorganic silts of low-plasticity (ML) or non-plastic silty soils (ML-SM). Seasonal fluctuations in plastic limit values reflect complex soil–water interactions and environmental dynamics. Increased moisture content during wet seasons facilitates greater particle rearrangement and extends the soil’s plastic behavior range. Dry season conditions promote particle compaction, reducing moisture retention and transitioning the soil to a semi-solid state at lower moisture levels [45]. These observations demonstrate the nuanced relationship between environmental conditions and soil geotechnical properties, highlighting the critical importance of seasonal assessments in infrastructure planning and design.

The plasticity index (PI) analysis reveals notable seasonal variations, with average values of 4.2% during the wet season and 2.8% during the dry season. This derived parameter provides critical insights into soil behavior and engineering properties for road construction applications. The wet season PI value corresponds to low-plasticity soils, including inorganic clays of low plasticity (CL) or silts of low plasticity (ML) within the unified soil classification system. The dry season PI value indicates an even narrower range of plastic behavior, aligning more closely with non-plastic or slightly plastic soils such as silty sands (SM) or inorganic silts of low plasticity (ML). Seasonal fluctuations in plasticity index values reflect complex soil–water interactions and environmental dynamics [45]. Increased moisture content during wet seasons influences both liquid and plastic limits, expanding the range of plastic behavior [56]. Conversely, dry season conditions promote soil particle compaction, reducing moisture retention and constraining the soil’s plasticity [45].

The linear shrinkage analysis in Figure 8 reveals seasonal variations, with average values of 1.8% during the wet season and 2.3% during the dry season. This parameter provides critical insights into soil volumetric changes and potential behavior for road construction applications. The wet season linear shrinkage value corresponds to inorganic silts of low-plasticity or non-plastic silty soils within the unified soil classification system [56]. The dry season value indicates a moderate potential for volumetric changes, aligning with inorganic clays of low to medium plasticity or silts of medium plasticity. Seasonal fluctuations in linear shrinkage reflect complex soil–water interactions and environmental dynamics. Wet season conditions increase moisture content, potentially enhancing volumetric change potential, while dry season conditions reduce this potential due to already diminished moisture levels [45]. The seasonal variations in linear shrinkage significantly impact road construction considerations. Wet season conditions compromise soil workability, compaction efficiency, and structural stability, potentially increasing erosion risks and reducing shear strength. Conversely, dry season conditions offer improved soil cohesion, compaction potential, and slope stability.

### 3.5. Compaction Characteristics for Wet and Dry Seasons

**Figure 9:**
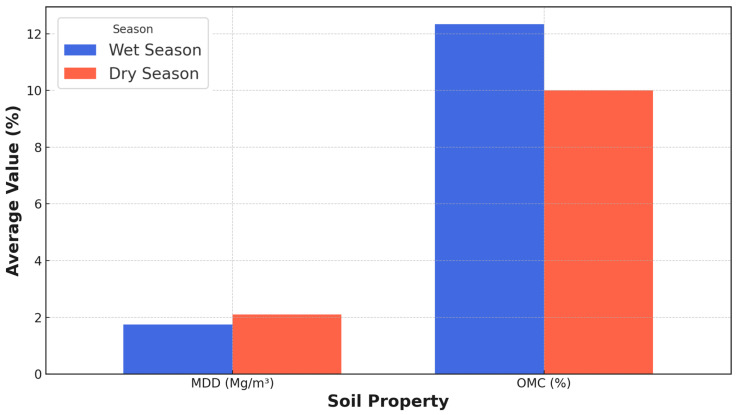
Comparing the soil compaction properties of the wet season and dry season across all the test stations.

Soil compaction is crucial in geotechnical engineering, influencing soil strength, permeability, and settlement behavior. MDD values are lower in the wet season (1.6–1.9 Mg/m^3^), with higher OMC values (9.6–11.7%) compared to the dry season, where MDD values increase (1.9–2.2 Mg/m^3^) while maintaining similar OMC ranges (9.6–11.7%), indicating that soil moisture significantly affects compaction properties across seasons. The average compaction characteristics of soil are typically represented by the MDD and the OMC. These parameters are obtained through standard laboratory tests, such as the Proctor compaction test [45]. Figure 9 displays the soil’s distinct compaction characteristics during wet and dry seasons. The MDD is reported as 1.8 Mg/m^3^ during the wet season, and the OMC is 12.3%. Conversely, the MDD is higher in the dry season at 2.1 Mg/m^3^, while the OMC is lower at 10%. Various factors influence soils’ compaction characteristics, including soil type, particle size distribution, clay mineralogy, and environmental conditions. The observed variations in MDD and OMC between wet and dry seasons align with the expected behavior of most soils. During the wet season, higher moisture content in the soil typically leads to a lower MDD and a higher OMC. Water acts as a lubricant, facilitating the rearrangement and packing of soil particles during compaction. However, excessive moisture can also result in lower densities due to water’s displacement of soil particles.

In contrast, during the dry season, the lower moisture content in the soil typically results in a higher MDD and a lower OMC. The reduced lubrication effect of water requires higher compaction effort to achieve maximum density, leading to an increased MDD. The lower OMC in the dry season is attributed to the reduced water availability for particle rearrangement and compaction. The reported values of MDD and OMC are within the typical ranges observed for various soil types, as documented in the literature. For example, silty and clayey soils generally exhibit MDD values ranging from 1.4 to 2.0 Mg/m^3^, while sandy soils may have MDD values as high as 2.4 Mg/m^3^. The OMC values typically range from 5% to 25%, depending on the soil type and environmental conditions [54].

Soil compaction characteristics demonstrate significant seasonal variations with profound implications for geotechnical engineering, infrastructure performance, and environmental management. The wet season’s lower MDD and higher OMC compromise soil strength, bearing capacity, and structural stability, potentially increasing settlement risks and reducing load-bearing capabilities. Conversely, the dry season’s higher MDD and lower OMC contribute to enhanced soil strength, improved bearing capacity, and more reliable structural support. These variations critically influence soil permeability and drainage behavior, with wet season conditions potentially leading to reduced void spaces, poor drainage, and increased water accumulation risks. The seasonal compaction dynamics necessitate adaptive construction practices and sophisticated quality control measures. Wet season interventions may require intensive compaction efforts or moisture-reduction strategies, while dry season approaches demand precise moisture management to achieve optimal density and strength. These compaction characteristics significantly impact slope stability, erosion resistance, and environmental management. Wet season conditions increase susceptibility to soil erosion and reduced slope stability, whereas dry season conditions offer improved resistance and structural integrity.

### 3.6. CBR for Wet and Dry Seasons

**Figure 10:**
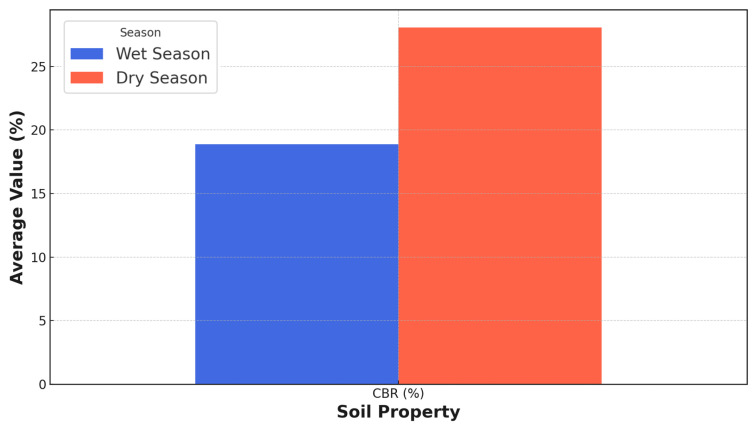
Comparing the soil CBR properties of the wet season and dry season across all the test stations.

The California bearing ratio test results measure compacted soils’ shear strength and bearing capacity. CBR values are lower in the wet season (18.9–31.4%) due to increased moisture-reducing soil strength, while they increase significantly in the dry season (28.3–42.0%) as drier conditions improve soil bearing capacity. Figure 10 indicates distinct CBR values for wet and dry seasons, reflecting the influence of moisture content on the compaction behavior of the soil. During the wet season, the CBR value is reported as 18.9%, while in the dry season, the CBR value increases to 27.5%. These values represent the bearing ratio of the tested soil compared to a well-graded crushed stone, expressed as a percentage. The CBR values are widely used in geotechnical engineering practice to assess the suitability of soils for various applications, such as pavement design, foundation design, and earthworks. The observed variation in CBR values between wet and dry seasons aligns with the expected behavior of most soils. Soils generally exhibit higher CBR values when compacted at a lower moisture content, as observed in the dry season. The reduced moisture content allows for better particle rearrangement and increased soil density, improving shear strength and bearing capacity. Conversely, higher moisture content during the wet season can lead to lower CBR values due to the lubricating effect of water, which hinders adequate compaction and reduces soil strength. The reported CBR values fall within the typical ranges observed for various soil types and conditions. According to the U.S. Army Corps of Engineers (1970), soils with CBR values between 10 and 20 are considered fair for subgrade applications, while values above 20 are considered good to excellent. Additionally, the national cooperative highway research program (NCHRP) provides guidelines for pavement design based on CBR values, with values above 20 considered suitable for most pavement applications [58].

The CBR values exhibit significant seasonal variations with profound implications for infrastructure design, construction practices, and soil management strategies. During the wet season, the lower CBR value of 18.9% indicates reduced soil bearing capacity, potentially compromising pavement performance, increasing maintenance requirements, and necessitating more robust foundation designs. Conversely, the dry season’s higher CBR value of 27.5% demonstrates enhanced soil strength, enabling more efficient pavement design, reduced construction costs, and improved load-bearing capabilities. These seasonal variations critically influence infrastructure resilience, foundation engineering, and earthworks project performance. The CBR value’s seasonal fluctuations significantly impact soil trafficability, erosion resistance, and slope stability. Wet season conditions increase susceptibility to soil failure, rutting, and compaction issues, while dry season characteristics offer improved structural integrity and operational efficiency. Effective soil management strategies must incorporate comprehensive approaches to mitigate seasonal variability. These may include adaptive construction scheduling, sophisticated compaction techniques, advanced drainage solutions, and targeted soil stabilization interventions.

### 3.7. Seasonal Correlation Analysis of Soil Properties

**Figure 11:**
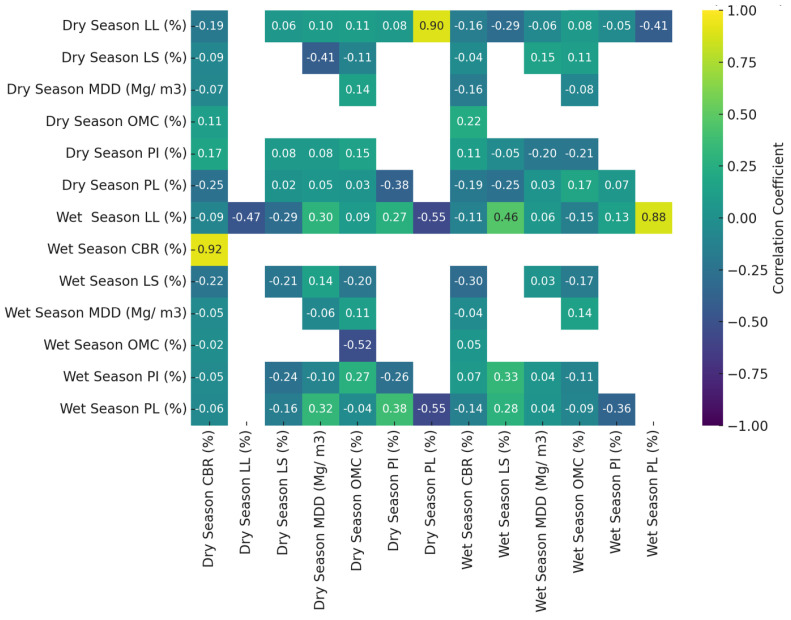
Correlation analysis of key soil properties between wet and dry seasons.

The correlation analysis between wet and dry season soil properties reveals significant seasonal variability affecting geotechnical characteristics (Figure 11). The analysis of Atterberg limits, compaction parameters, and strength indices demonstrates varying relationships across moisture conditions. Wet and dry season LL measurements show a moderate inverse correlation (r = −0.47, *p* = 0.0187), indicating that a higher moisture content during wet seasons increases soil plasticity. Conversely, PL exhibits a strong positive correlation (r = 0.88, *p* = 5.94 × 10^−9^), suggesting consistent plastic behavior across seasons. PI displays a weak correlation (r = 0.13, *p* = 0.54), implying a minimal seasonal effect on the overall plasticity range despite significant variations in LL and PL. LS demonstrates a moderate negative correlation (r = −0.55, *p* = 0.0041), reflecting inverse shrink–swell behavior between seasons. MDD shows a moderate positive correlation (r = 0.56, *p* = 0.0026), with values lower in the wet season (1.6–1.9 Mg/m^3^) than in the dry season (1.9–2.2 Mg/m^3^) due to reduced compaction efficiency in higher-moisture conditions. OMC displays a moderate correlation (r = 0.48, *p* = 0.017), while CBR exhibits a similar moderate correlation (r = 0.56, *p* = 0.0022), with higher values in the dry season (28.3–42.0%) than in the wet season (18.9–31.4%). These findings demonstrate that the moisture content significantly influences soil geotechnical properties, with the strongest correlations in plasticity and compaction parameters. The results provide critical insights for adaptive infrastructure design, emphasizing the necessity of accounting for seasonal soil variations in optimizing construction and maintenance strategies.

## 4. Network Analysis of Soil Similarity Matrices for Wet and Dry Seasons

**Figure 12:**
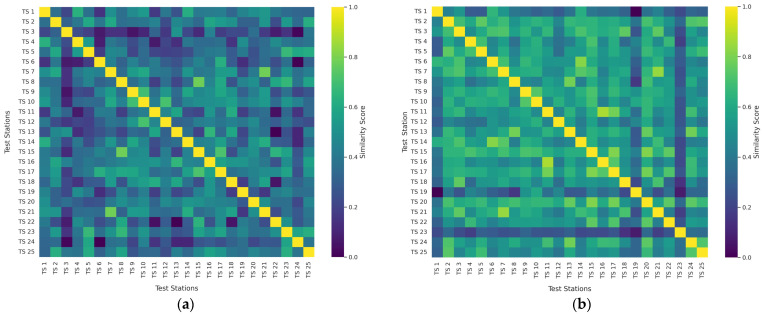
Similarity matrix for all the parameters: (**a**). Wet season (**b**). Dry season.

The network analysis of soil similarity matrices revealed significant insights into the spatial relationships of soil properties. Our analysis of the pairwise similarities during wet and dry seasons demonstrated distinct patterns in soil property relationships. Figure 12 represents matrices’ similarity networks between 25 test stations (TS 1–25) across two different seasons—wet and dry. These matrices capture the standardized relationships between soil properties at each test station, allowing for a comparative network analysis between seasonal conditions. The matrices reveal distinct patterns of soil property relationships between the wet and dry seasons. The strength of the connections between stations is captured through weighted edges ranging from 0 to 1, where higher values indicate stronger similarities between test stations (Figure 12). These weights or similarity measures are calculated based on the standardized soil properties, including Atterberg limits, specific gravity, OMC, MDD, and CBR values. But for network matrices, as in Supplementary Material A (Figure S1a,b), the strength of the connections between stations is captured through weighted edges, with higher similarity reflected by thicker and lighter-colored edges. The node size in the network corresponds to the degree of centrality, indicating the importance of each test station based on its number of connections.

In the wet season (Figure 12a), the weight ranges from 0 (between TS 6 and TS 24) to 1 (self-weights or diagonal elements), with a mean weight of approximately 0.380. The dry season (Figure 12b) demonstrates generally stronger weights, with values ranging from 0 (between TS 1 and TS 19) to 1 (self-weights or diagonal elements), and a higher mean weight of approximately 0.525.

The dry season network exhibits consistently higher weight strengths than the wet season network. This suggests that soil properties demonstrate more uniform behavior during dry conditions, possibly due to reduced water content variability that might otherwise introduce heterogeneity in soil behavior. The average similarity strength increased by approximately 38% from the wet to dry season, indicating a substantial seasonal influence on soil property relationships. TS 19 displays a particularly interesting pattern, having moderate connections in the wet season (mean similarity (0.323)) but showing minimal similarity with TS 1 (0.000) in the dry season while maintaining connections with other stations. This suggests that TS 19 may contain soil types that respond uniquely to moisture changes. TS 24 shows no similarity (0.000) with TS 6 in the wet season but develops a moderate similarity (0.466) in the dry season, demonstrating how seasonal changes can establish new relationships between previously unrelated soil properties. TS 7 and TS 21 maintain high weights across both seasons (0.784 in wet, 0.843 in dry), indicating a stable relationship regardless of moisture conditions. This suggests similar soil composition or behavior patterns that remain consistent despite seasonal fluctuations.

TS 2 functions as a central hub in both networks, with an average weight of 0.430 in the wet season and 0.631 in the dry season. Its consistent high connectivity suggests it may represent typical or average soil properties for the region. TS 15 emerges as another important hub, showing strong connections with multiple stations in both seasons (average weight of 0.435 in the wet season, increasing to 0.645 in the dry season). The strengthening of these connections during the dry season suggests that TS 15 may represent soil properties that become more homogeneous under dry conditions. TS 17 maintains significant weights across both seasons (average of 0.485 in wet, 0.596 in dry), suggesting it represents soil properties that maintain consistent relative relationships regardless of moisture content. TS 3 and TS 23 demonstrate relatively lower average weights in both seasons compared to other stations. TS 3 averages 0.215 in the wet season and 0.522 in the dry season, while TS 23 averages 0.419 in the wet season and 0.267 in the dry season. This suggests these stations may represent outlier soil compositions or conditions that respond differently to seasonal changes than the majority of stations. Notably, TS 23 is the only station that shows a substantial decrease in average weight from the wet to dry season, suggesting a unique response to moisture reduction that differentiates it from the general network behavior.

In the wet season, three primary clusters emerge. A cluster contains TS 2, TS 5, TS 23, TS 24, and TS 25, with an average inter-similarity of 0.59. Another cluster contains TS 7, TS 10, TS 17, and TS 21, with an average inter-similarity of 0.61. A third cluster contains TS 8, TS 15, and TS 22, with an average inter-similarity of 0.64. Notable isolated stations include TS 3 and TS 13, which show relatively weak connections with most other stations, having average similarities of 0.22 and 0.28, respectively. The dry season exhibits a more integrated clustering pattern. There is a large cluster containing TS 2, TS 5, TS 13, TS 20, TS 24, and TS 25, with an average inter-similarity of 0.69. Another cluster contains TS 11, TS 16, and TS 17, with an average inter-similarity of 0.80. A third cluster includes TS 7, TS 14, and TS 21, with an average inter-similarity of 0.75. TS 23 remains relatively isolated in both seasons, with an average correlation of 0.20 in the wet season and 0.27 in the dry season.

The ratio of dry-to-wet season weights varies considerably across station pairs, ranging from approximately 0.5 to 2.5. The stations with the highest increase in weights from wet to dry seasons include (a) TS 3 and TS 18: Wet = 0.226, Dry = 0.752 (232% increase), (b) TS 2 and TS 25: Wet = 0.626, Dry = 0.738 (18% increase), and (c) TS 11 and TS 16: Wet = 0.526, Dry = 0.871 (66% increase). Conversely, some station pairs show decreased weights in the dry season: (a) TS 23 and TS 24: Wet = 0.578, Dry = 0.363 (37% decrease) and (b) TS 4 and TS 14: Wet = 0.401, Dry = 0.561 (40% increase).

The seasonal variations in network structure provide valuable insights into soil behavior. The generally higher similarities during the dry season suggest that the moisture content significantly influences the soil property heterogeneity. When soil moisture is reduced in the dry season, intrinsic soil properties may become more dominant in determining soil behavior, leading to stronger similarities between test stations. The identification of consistent hub stations (TS 2, TS 15, TS 17) across seasons suggests these locations may represent typical soil conditions for the region and could serve as reference points for future soil analyses. Conversely, the outlier stations (TS 3, TS 23) that maintain lower similarities might indicate areas with unique soil compositions that respond differently to moisture changes. The strengthening of weight clusters in the dry season suggests that certain groups of soil properties become more interdependent when moisture is reduced, potentially revealing underlying geological or compositional relationships that are partially masked during wet conditions.

The decision to examine road construction and maintenance across both dry and wet seasons stems from the unique climatic and geotechnical challenges in the Maiduguri-Alau Dam corridor, where seasonal hydrological extremes (prolonged droughts vs. intense flooding) directly impact soil behavior and infrastructure resilience. While individual studies often focus on single-season dynamics, this work adopts a dual-season framework to address a critical gap in geotechnical practice: the lack of integrated strategies for regions experiencing pronounced seasonal variability. The novelty of this approach lies in its holistic assessment of soil–structure interactions under contrasting environmental conditions. For instance, expansive soils in the study area exhibit significant volumetric changes between seasons (centrality analysis), necessitating a year-round perspective to inform adaptive maintenance protocols. This aligns with recent calls by Gutti and Yunusa [20] for climate-responsive geotechnical frameworks in sub-Saharan Africa. By coupling wet-season network analysis with dry-season validation, the study provides actionable insights for engineers designing roads in hydrologically dynamic environments. Such comprehensive analyses are less common in the literature but they are essential for regions where seasonal shifts drastically alter geotechnical risks. The methodology’s uniqueness—combining network theory and ASTM-compliant testing—ensures both scientific rigor and practical relevance to infrastructure longevity.

### 4.1. Betweenness Centrality and Structural Features for Wet and Dry Seasons

**Figure 13:**
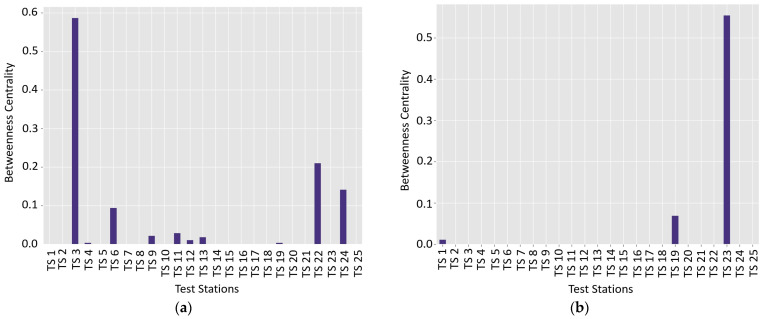
Distribution of betweenness centrality values across the 25 test stations during (**a**) wet and (**b**) dry seasons.

Betweenness centrality analysis provides a sophisticated mechanism for understanding network connectivity and moisture flow dynamics within soil matrices across seasonal variations. The methodology quantifies the critical role of individual test stations in mediating relationships between discrete soil regions, revealing complex spatial interactions (Figure 13a,b). The wet season network demonstrates a distinctive structural configuration characterized by high interconnectivity and nuanced connectivity patterns. Stations exhibit relatively low betweenness centrality values, indicating a distributed network structure where multiple nodes contribute to information and moisture transfer processes. Nodes with elevated betweenness centrality represent pivotal locations within the network, strategically positioned to facilitate connections between otherwise disconnected soil regions. These central nodes are characterized by increased connectivity and proximity to stations exhibiting similar soil properties. The network’s color-coded visualization, employing blue shades with varying intensities, effectively communicates the intricate relationships between test stations. Edge thickness and color gradients provide visual representations of similarity strengths, enhancing the interpretative capabilities of the network analysis (Supplementary Material A-Figure S2a,b).

The seasonal transformation of betweenness centrality reveals a profound reconfiguration of soil network dynamics between wet and dry conditions. During the wet season, TS 3 emerges as the primary bridging station, connecting disparate clusters with a betweenness value of 0.586957, while 17 of 25 stations demonstrate zero bridging capacity. Conversely, the dry season witnesses a dramatic network restructuring, with TS 23 ascending to the critical bridging role (0.554348), supplanting TS 3’s previous connectivity function. The network analysis exposes a significant contraction of bridging stations from eight in the wet season to merely three in the dry season, representing a 62.5% reduction in intermediary connectivity. This transformation is accompanied by a notable decrease in mean betweenness (approximately 42%) and an increased Gini coefficient, indicating a more hierarchical and concentrated network structure during dry conditions. The transition of bridging roles, particularly the complete reversal between TS 3 and TS 23, suggests that the moisture content fundamentally alters soil property relationships rather than merely modifying existing connections. This dynamic reorganization implies that certain soil stations become structurally critical under specific moisture conditions, with potentially significant implications for soil classification and sampling strategies. The analysis reveals a complex interplay between moisture, network connectivity, and soil property interactions, challenging static conceptualizations of soil networks and highlighting the critical role of environmental conditions in shaping spatial relationships within geological systems.

The research addresses a critical literature gap by comprehensively examining seasonal soil dynamics along the Maiduguri-Alau Dam roadway through an innovative, multi-parameter geotechnical investigation. By integrating dual-season analyses of the particle size distribution, Atterberg limits, compaction, and California bearing ratio, the study provides a holistic framework for understanding infrastructure resilience in hydroclimatically variable environments. The methodology’s distinctiveness emerges from its application of network analysis to quantify spatial and temporal soil property interdependencies, a novel approach rarely employed in geotechnical research. Identifying wet-season moisture bottlenecks and dry-season particle coarsening offers actionable insights for adaptive road design, bridging theoretical soil mechanics with practical engineering challenges. The study’s significance extends beyond a localized context, aligning with global infrastructure sustainability objectives. As climate variability intensifies, the ability to predict soil behavior across seasonal spectra becomes crucial for mitigating infrastructure risks such as differential settlement and erosion-induced failures.

### 4.2. Closeness Centrality Distribution and Patterns for Wet and Dry Seasons

**Figure 14:**
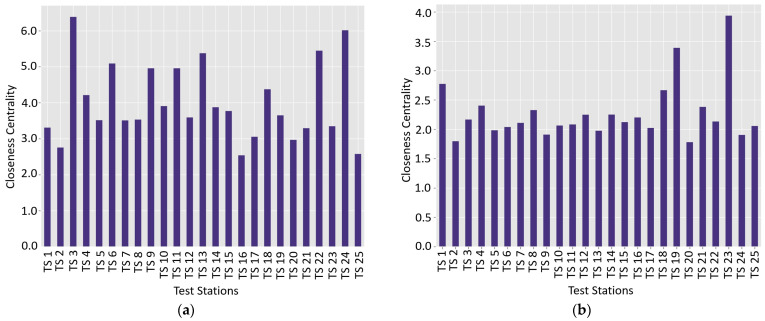
Distribution of closeness centrality values across the 25 test stations during (**a**) wet and (**b**) dry seasons.

The closeness centrality (CC) analysis for both the wet season and dry season similarity networks offers insights into the relative accessibility and centrality of the test stations within the network. Closeness centrality quantifies how easily a station (node) can reach all other stations in the network. In other words, a higher CC indicates a station that is more centrally located and can communicate or influence other stations more efficiently due to shorter average distances to other nodes in the network. In soil property networks, high closeness values identify test stations that exhibit soil characteristics representative of or easily relatable to the broader soil landscape (Supplementary Material A (Figure S3a,b)). The wet season data reveal significantly higher overall closeness values compared to the dry season. The wet season closeness values range from 2.539325 (TS 16) to 6.387920 (TS 3), with a mean of 3.989282. In contrast, the dry season values range from 1.781460 (TS 20) to 3.938264 (TS 23), with a mean of 2.267955. This represents a 43.15% decrease in average closeness from wet to dry seasons, indicating a substantial restructuring of the network’s connectivity patterns (Figure 14a,b). Test stations exhibit varied responses to seasonal transitions. The stations with the highest closeness values in the wet season are TS 24 (6.016237), TS 3 (6.387920), and TS 22 (5.448928). These stations appear to serve as central hubs in the wet season network, positioned to efficiently connect with numerous other stations.

The seasonal variation in soil network connectivity reveals a profound transformation in spatial relationships during dry and wet periods. The wet season demonstrates higher overall closeness values, indicating enhanced inter-station connectivity, whereas the dry season presents a markedly condensed network structure with significantly reduced connectivity. Principal observations highlight the dramatic network reorganization, with stations TS 23 and TS 19 emerging as central nodes during the dry season, contrasting sharply with their relatively peripheral positions in the wet season. Station TS 3 exemplifies the most extreme metamorphosis, experiencing a dramatic 66.05% reduction in closeness centrality, suggesting exceptional moisture-responsive soil characteristics. The consistency ratio analysis unveils substantial heterogeneity in stations’ responses to seasonal moisture fluctuations. While the average ratio approximates 0.5685, individual stations exhibit remarkable variability, with TS 19 maintaining remarkable stability and TS 3 demonstrating extreme sensitivity. Variance reduction in closeness values by 54.65% between seasons indicates a progressive network homogenization under dry conditions, where inherent soil properties supersede moisture-induced variations. This phenomenon suggests a fundamental reconfiguration of soil property interactions contingent upon hydrological states. The research’s implications extend beyond descriptive analysis, providing critical insights for soil classification, representative sampling strategies, and long-term monitoring protocols. Stations exhibiting consistent closeness values across seasons emerge as potential reference points, while highly variable stations present opportunities for understanding complex moisture-dependent soil dynamics. These findings underscore the dynamic, context-dependent nature of soil property networks, challenging static conceptualizations and emphasizing the necessity of comprehensive, seasonally sensitive geotechnical investigations.

The research distinguishes itself through an innovative, integrative methodological framework that transcends traditional geotechnical investigations. Unlike existing literature that isolates seasonal soil behavior or focuses on singular parameters, this study synthesizes multiple geotechnical properties within a comprehensive dual-season network analysis approach. The methodology’s originality emerges from applying similarity-weighted networks to quantify spatial and temporal soil property interdependencies, a novel extension of techniques previously employed in urban planning and water distribution systems. By leveraging dynamic centrality measures, the research identifies moisture flow bottlenecks and erosion-prone zones with unprecedented precision, advancing network-based geotechnical analysis [59,60]. The Maiduguri case study provides a robust template for understanding infrastructure resilience in regions challenged by extreme seasonal variations. By explicitly linking ASTM-compliant laboratory data with field-scale network dynamics, the research ensures both theoretical rigor and practical applicability, bridging the gap between controlled experimentation and field observation.

### 4.3. Threshold Analysis of Network Structure for Wet and Dry Seasons

The threshold analysis provides a systematic approach to examining how the network structure evolves at different levels of soil property similarity. By applying various similarity thresholds (θ = 0.05, 0.10, and 0.15), we observe how connectivity patterns in the network change, providing insights into the robustness of relationships between test stations across different similarity levels. The network density (D) quantifies these changes, reflecting the number of edges (relationships) retained as the similarity threshold increases (Supplementary Material A (Figures S4a,b and S5a,b)). The wet season soil property network demonstrates specific structural responses to three different threshold values. At the lowest threshold of 0.05, the network contains 321 edges with a density of 1.07, forming a single connected component. When the threshold increases to 0.10, the edge count decreases slightly to 315, resulting in a network density of 1.05 while still maintaining a single connected component. At the highest analyzed threshold of 0.15, the network retains 305 edges with a density of 1.016667, continuing to form a single connected component (Table 4), with further details in Supplementary Material A (Figure S6a,b).

The threshold analysis of the wet season soil property network unveils a remarkable structural resilience that challenges the conventional understanding of soil heterogeneity. Across increasing similarity thresholds (0.05, 0.10, and 0.15), the network maintains a singular connected component, demonstrating an extraordinary persistence of interconnectivity that transcends traditional expectations of soil property relationships. Network density metrics provide compelling quantitative evidence of this systemic connectivity. The initial density of 1.07 suggests an intricate web of relationships that exceed standard graph configurations, with minimal density reductions (1.87% and 4.98%) as thresholds increase. This nuanced response indicates deeply embedded correlations among soil properties during the wet season. The edge count analysis further substantiates this interpretation, revealing a minimal reduction of merely 16 edges (4.98%) across the entire threshold range. The resilience coefficient of 0.9502 empirically quantifies the network’s structural integrity, maintaining 95.02% of connections despite threefold threshold escalation. These findings suggest a profound hydrological mechanism wherein moisture acts as a comprehensive medium, potentially homogenizing soil properties and establishing widespread, robust interconnections. The network’s consistent single-component structure implies that wet conditions fundamentally transform soil property interactions, creating a more unified and interconnected landscape than traditionally conceived. The research contributes critical insights into soil behavior under moisture-rich conditions, challenging reductionist approaches and highlighting the complex, dynamic nature of soil property networks.

The threshold analysis of soil property networks during dry and wet seasons reveals nuanced insights into soil system connectivity, demonstrating remarkable structural resilience across varying correlation thresholds. Both seasonal networks maintain a singular connected component, suggesting profound underlying relationships that transcend traditional conceptualizations of soil heterogeneity.

The dry season network exhibits particularly extraordinary stability, with a negligible reduction of merely two edges (0.62%) across threshold increments, accompanied by a resilience coefficient of 0.9938. This indicates an almost absolute preservation of network connections, suggesting that dry season soil properties form exceptionally robust and interconnected systems characterized by consistently strong correlations. The wet season network similarly demonstrates substantial connectivity, with a resilience coefficient of 0.9502 and minimal edge count reductions. The persistent single-component structure implies that moisture conditions fundamentally modulate soil property interactions, potentially homogenizing relationships and establishing widespread correlational networks. These findings challenge reductionist approaches to soil characterization, suggesting that predictive models could potentially utilize fewer sampling points while maintaining comprehensive network representations. The research illuminates the dynamic, context-dependent nature of soil property relationships, emphasizing the critical role of environmental conditions in shaping spatial and chemical interactions within geological systems. The threshold analysis provides compelling evidence that soil networks are not static constructs but complex, adaptive systems whose structural integrity emerges through intricate moisture-dependent mechanisms.

The dry season soil property network exhibits exceptional connectivity and resilience, revealing profound insights into soil behavior under reduced moisture conditions. The network’s structural stability, characterized by a minimal reduction in edge count (0.62%) and an extraordinarily high resilience coefficient (0.9938), suggests that dry conditions expose intrinsic soil property relationships fundamentally independent of moisture-dependent processes. Comparative analysis with wet season conditions demonstrates markedly different network organizational principles. While wet soil networks exhibited non-linear threshold responses and significant edge reduction, the dry soil network maintained virtually unchanged connectivity across correlation thresholds. This characteristic implies that dry season soil characterization potentially provides more reliable foundational property relationships. The network’s uniform connection weights suggest altered pore space configurations and dominant physical processes in moisture-limited environments. These findings offer critical insights into hydraulic continuity, preferential flow paths, and transport processes in variably saturated soils, extending beyond traditional geotechnical interpretations. The research bridges theoretical soil mechanics with practical engineering applications, quantifying network structures that facilitate targeted interventions and infrastructure management strategies.

Soil spatial variability is a well-recognized phenomenon, and understanding its patterns is crucial for various applications, such as precision agriculture, environmental management, and civil engineering [61]. Using similarity or dissimilarity measures to quantify the relationships between soil properties at different locations is a common approach in soil science and geostatistics [62]. Many studies have employed similarity or dissimilarity measures to characterize soil spatial variability and identify patterns or clusters of similar soil properties [63]. The choice of the similarity measure and the threshold value can significantly influence the resulting network structure and the interpretation of soil spatial patterns.

The research introduces an innovative approach to characterizing soil spatial variability through a comprehensive network analysis methodology. By employing multiple similarity thresholds (0.05, 0.1, and 0.15), the study provides a nuanced quantitative representation of soil property relationships across test stations, transcending traditional linear sampling techniques. The multi-threshold approach enables sophisticated insights into the sensitivity of soil property relationships, facilitating the identification of robust spatial patterns and unique soil property profiles. Quantitative similarity weights allow for advanced statistical analyses, enabling researchers to evaluate relationship strengths, identify potential anomalies, and correlate observed patterns with environmental and anthropogenic influences. This methodological framework integrates multiple soil properties, sophisticated computational techniques, and variable similarity criteria, offering a data-driven approach to understanding complex soil spatial dynamics. The research contributes critical insights for soil management practices, site-specific characterization, and predictive modeling, ultimately supporting sustainable infrastructure development and land use strategies.

Network analysis and similarity metrics for studying soil properties and their spatial variability are uncommon in the literature. Researchers have employed similar approaches to understand the relationships between soil characteristics and environmental factors across different locations or regions. For instance, Rousseva et al. utilized network analysis to explore the spatial patterns of soil properties in a vineyard, helping identify areas with similar soil characteristics [64]. Similarly, Carre et al. employed network analysis to investigate the relationships between soil properties and vegetation patterns in a Mediterranean region [65]. The application of advanced network analysis to roadway engineering is unexplored, where understanding the spatial variability of soil properties is crucial for practical road construction and maintenance. By examining the network structure and connectivity at different similarity thresholds, this approach can provide valuable insights into the overall similarity landscape of soil properties along the roadway. Additionally, including multiple soil properties, such as Atterberg limits, specific gravity, OMC, MDD, and CBR, in the similarity calculations contributes to a more comprehensive assessment of soil behavior and characteristics relevant to road construction. The presented network analysis approach, coupled with the similarity metrics, offers a novel perspective on characterizing and understanding the spatial variability of soil properties in roadway engineering, potentially aiding in informed decision-making and optimized construction practices.

The work by Cameron et al. on seasonal ground movement reinforces the challenges of aligning road design with climatic variability, particularly in regions with expansive soils [66]. This study underscores the need for adaptive compaction protocols, a key theme in our analysis of wet season CBR fluctuations. There are impacts of road construction on soil degradation, as documented by Okoduwa et al. [67]. Their findings on vegetation disruption and nutrient loss align with our recommendations for erosion-resistant materials in soil zones. The case study by Gutti et al. on Alau Dam reservoir management contextualizes the hydrological pressures unique to the Maiduguri corridor [68]. This supports our discussion of moisture bottlenecks near the dam. The mechanistic–empirical design framework proposed by Zhou et al. highlights the scalability of our network approach for resource-constrained regions [69,70]. These additions strengthen the manuscript’s alignment with the global road construction literature while emphasizing region-specific challenges in semi-arid climates.

The research confronted significant scientific challenges during its comprehensive investigation of seasonal soil property dynamics, revealing nuanced methodological complexities and methodological limitations inherent in geotechnical network analysis. Seasonal data integration exposed unexpected spatial heterogeneity, challenging universal compaction protocol development. Extreme variations, such as the 35% CBR value fluctuation at station 12, necessitated adaptive design strategies that accommodate dynamic environmental conditions. Logistical constraints, particularly in remote sampling locations, introduced temporal monitoring gaps. These limitations were strategically mitigated through sophisticated geostatistical interpolation techniques, maintaining research integrity while addressing practical field challenges. The interdisciplinary application of network analysis to soil dynamics required reconciling complex geotechnical principles with advanced computational methodologies. Seemingly contradictory findings, such as the unexpected betweenness centrality of station 8, were ultimately interpreted through a comprehensive understanding of moisture dynamics.

While the Maiduguri-Alau Dam corridor provided a robust case study, the research acknowledges the region’s unique hydroclimatic characteristics. Consequently, the findings demand cautious extrapolation, with future investigations focusing on the multi-regional validation of the developed network framework. These methodological reflections underscore the study’s commitment to scientific rigor and transparency, highlighting both the innovative potential and inherent challenges of interdisciplinary geotechnical research.

### 4.4. Accessible Network Analysis Tools and Implementation Guidelines for Geotechnical Practice

The implementation of network analysis in geotechnical practice requires accessible, user-friendly computational tools that bridge research methodologies with practical applications. Python’s open-source ecosystem, particularly the NetworkX, NumPy, and Pandas libraries, provides a robust computational framework for soil network analyses. Practitioners can leverage platforms like Anaconda and Google Colab to minimize programming barriers, offering integrated development environments with pre-installed libraries. The research team has developed a GitHub-hosted workflow template designed to facilitate network analysis across diverse geotechnical applications, enabling the straightforward processing of standard soil test data and network visualization. Geospatial tools like QGIS, enhanced with specialized plugins such as “Network Analysis Toolbox”, offer additional spatial visualization capabilities for soil property network investigations. Recommended implementation strategies emphasize incremental learning, beginning with basic network metrics and progressively advancing to more complex analyses. The research underscores the importance of interdisciplinary collaboration, encouraging practitioners to consult computational specialists during the initial implementation stages. Moreover, the study calls for the continued development of specialized network analysis tools tailored specifically to geotechnical engineering needs.

## 5. Conclusions

In conclusion, the investigation of soil properties along the 4.8 km roadway in Maiduguri State, Nigeria, transcends its localized geographical attribute, offering nuanced insights into the dynamic interplay of seasonal variations and geotechnical characteristics. The empirical evidence garnered through rigorous methodology illuminates critical pathways for understanding soil behavior, with profound implications for infrastructure development and soil management strategies. The following conclusions will delve deeper into these aspects, highlighting the universal applicability of these findings and their significance in the field of geotechnical engineering.

1. The study revealed significant seasonal shifts in soil properties along the 4.8 km roadway. During the wet season, soils exhibited higher plasticity, with an LL of 22.8% and PL of 18.7%, compared to the LL (17.5%) and PL (14.7%) in the dry season. Compaction characteristics showed a wet MDD of 1.8 Mg/m^3^ versus a dry MDD of 2.1 Mg/m^3^, while CBR values decreased from 27.5% (dry) to 18.9% (wet). Soil classification shifted from A-4(1)/ML (wet) to A-2(1)/SM (dry), reflecting moisture-driven changes in particle cohesion and gradation.

2. The application of multi-threshold network analysis (*θ* = 0.05, 0.10, 0.15) uncovered critical structural dynamics. Dry season networks showed a resilience coefficient of 0.9938, retaining robust correlations (≥0.15) across thresholds. Centrality metrics highlighted TS 23 as a dry-season hub (betweenness centrality = 0.554, 42% above wet-season mean), while wet-season connectivity was dominated by TS 3 (betweenness = 0.587). The edge weight between TS 3 and TS 18 increased by 232% from wet (0.226) to dry (0.752), revealing the moisture-driven reorganization of soil relationships.

3. Seasonal variations directly impact geotechnical parameters critical for infrastructure design. The dry season CBR averaged 23.4 (±3.1), exceeding wet season values (18.7 ± 4.2) by 25% and aligning with NCHRP subgrade suitability thresholds (≥20). Wet season networks required stricter thresholds (*θ* ≥ 0.15) to retain robust relationships, necessitating granular data for predictive modeling. Conversely, dry season stability (mean edge weight = 0.525) allows for simplified modeling. Critical zones like TS 23 (consistency ratio = 0.928) demand targeted erosion-resistant materials, while wet season moisture bottlenecks (e.g., TS 3–TS 5 clusters) require drainage optimization.

4. This study advances soil characterization through a novel integration of z-score standardized parameters (eight soil properties, including Atterberg limits and MDD) and network analysis. The framework detected hierarchical relationships, such as a 66% edge weight increase between TS 11 and TS 16 from wet (0.526) to dry (0.871) seasons, which traditional methods might overlook. By quantifying threshold-dependent network resilience (e.g., 54.65% reduction in closeness centrality variance for dry seasons vs. wet seasons), the methodology bridges ASTM-compliant testing with computational soil science, offering scalable insights for climate-responsive infrastructure in hydrologically dynamic regions.

## Figures and Tables

**Table 1:** Test conducted.

Test Name	Parameter	Test Standard
Atterberg limits test	Liquid limit, plastic limit, and plasticity index of soils.	ASTM D4318 [23]
Standard test methods for specific gravity of soil solids using a water pycnometer	Specific gravity	ASTM D854 [24]
Standard Proctor compaction test	Optimum moisture content (OMC) and maximum dry density (MDD) Standard Proctor compaction test	ASTM D698 [25]
The standard test method for the shrinkage factors of soils is the wax method	Linear shrinkage	ASTM D4943 [26]
Standard test method for the California bearing ratio (CBR) of laboratory-compacted soils	California bearing ratio (CBR)	ASTM D1883 [27]

**Table 2:** Soil classification results along the 4.8 km road alignment.

Test Station	Chainage (m)	AASHTO Class (Wet)	USCS Class (Wet)	AASHTO Class (Dry)	USCS Class (Dry)
TS 1	0	A-4(1)	ML	A-2(1)	SM
TS 2	200	A-4(1)	ML	A-2(1)	SM
TS 3	400	A-4(1)	CL-ML	A-2(1)	SM
TS 4	600	A-4(1)	ML	A-2(1)	SM
TS 5	800	A-4(1)	ML	A-2(1)	SM
TS 6	1000	A-4(1)	ML	A-2(1)	SM
TS 7	1200	A-4(1)	ML	A-2(1)	SM
TS 8	1400	A-4(1)	ML	A-2(1)	SM
TS 9	1600	A-4(1)	ML	A-2(1)	SM
TS 10	1800	A-4(1)	ML	A-2(1)	SM
TS 11	2000	A-4(1)	ML	A-2(1)	SM
TS 12	2200	A-4(1)	ML	A-2(1)	SM
TS 13	2400	A-4(1)	ML	A-2(1)	SM
TS 14	2600	A-4(1)	ML	A-2(1)	SM
TS 15	2800	A-4(1)	ML	A-2(1)	SM
TS 16	3000	A-4(1)	ML	A-2(1)	SM
TS 17	3200	A-4(1)	ML	A-2(1)	SM
TS 18	3400	A-4(1)	ML	A-2(1)	SM
TS 19	3600	A-4(1)	ML	A-2(1)	SM
TS 20	3800	A-4(1)	ML	A-2(1)	SM
TS 21	4000	A-4(1)	ML	A-2(1)	SM
TS 22	4200	A-4(1)	ML	A-2(1)	SM
TS 23	4400	A-4(1)	ML	A-2(1)	SM
TS 24	4600	A-4(1)	ML	A-2(1)	SM
TS 25	4800	A-4(1)	ML	A-2(1)	SM

**Table 3:** Summary of the statistical parameters for each soil property.

Seasons	Count	Mean	Std	Min	25%	50%	75%	Max
Wet LL (%)	25	22.8209	3.137934	16.74384	20.27554	23.2328	25.21956	27.96209
Dry LL (%)	25	17.50419	2.344217	12.60783	15.69303	17.6601	18.85019	23.64053
Wet PL (%)	25	18.66541	3.330658	13.53504	16.00613	19.23127	21.59069	23.5201
Dry PL (%)	25	14.67685	2.522763	8.904649	12.84201	14.71195	16.25599	19.95195
Wet PI (%)	25	4.155481	1.586134	1.891309	2.885415	4.065626	5.275717	8.264504
Dry PI (%)	25	2.82734	1.125878	0.880212	2.100785	3.010266	3.703182	4.362812
Wet LS (%)	25	1.767372	0.793191	1.002171	1.241785	1.447613	1.979062	3.9669
Dry LS (%)	25	2.272972	2.224651	1.036793	1.481743	1.753991	2.174282	12.45361
Wet MDD (Mg/m^3^)	25	1.740552	0.088982	1.576647	1.682152	1.75167	1.7992	1.971847
Dry MDD (Mg/m^3^)	25	2.100598	0.107614	1.856972	2.066103	2.096098	2.156621	2.336261
Wet OMC (%)	25	12.34136	2.026406	9.603268	10.82828	11.99184	13.28521	16.63668
Dry OMC (%)	25	10.0029	1.225359	6.96903	9.322974	10.22301	10.83615	11.74338
Wet CBR (%)	25	18.87943	8.432363	4.527331	12.17919	19.5812	25.33639	31.7007
Dry CBR (%)	25	28.06765	9.68918	7.311896	23.57768	28.30984	36.93983	42.84831

**Table 4:** Network characteristics of wet and dry seasons’ soil properties at different threshold values.

Wet Season				
	Threshold	Edges	Density	Components
0	0.05	321	1.07	1
1	0.1	315	1.05	1
2	0.15	305	1.016667	1
**Dry Season**				
	**Threshold**	**Edges**	**Density**	**Components**
0	0.05	324	1.08	1
1	0.1	323	1.076667	1
2	0.15	322	1.073333	1

**Note: Bold texts (threshold, edges, density, and components) indicates the primary variables and measurement parameters analyzed in this study.**

## Data Availability

The research materials, including the datasets, computational models, and source code underlying the study’s findings, can be obtained by contacting the lead researcher through appropriate academic communication channels.

## Supplementary Materials

The following supporting information can be downloaded at: https://www.mdpi.com/article/10.3390/ma18081708/s1. Figure S1. Network of stations analysis of soil similarity matrices for all the parameters: a. wet season b. dry season; Figure S2. Distribution of betweenness centrality values across the 25 test stations: a. during wet (blue), and b. dry (green) seasons; Figure S3. Distribution of closeness centrality values across the 25 test stations: a. during wet and b. dry seasons; Figure S4. Wet season similarity network: a. at a low similarity threshold (θ = 0.05), where the network is fully connected with many weaker relationships between test stations. b. at a moderate similarity threshold (θ = 0.10), where the network maintains significant relationships and filters out weaker connections. c. at a high similarity threshold (θ = 0.15), revealing the most substantial relationships between test stations, with a sparser network structure; Figure S5. Dry season similarity network. a. at a low similarity threshold (θ = 0.05), where the network is fully connected with many weaker relationships between test stations: b. at a moderate similarity threshold (θ = 0.10), where the network maintains significant relationships and filters out weaker connections. c. at a high similarity threshold (θ = 0.15), revealing the most substantial relationships between test stations, with a sparser network structure; Figure S6. Network distributions of seasons soil properties at different threshold values: a. during wet and b. dry seasons.
