# Supplementary material for: Seasonal Dynamics in Soil Properties Along a Roadway Corridor: A Network Analysis Approach
Source: Materials (Basel). 2025 Apr 9;18(8):1708. doi: 10.3390/ma18081708 (PMC12028596; doi:10.3390/ma18081708)
Supplement: Supplementary file 1 [file materials-18-01708-s001.zip › materials-3517054-Supplementary.pdf]

Supplementary Material A

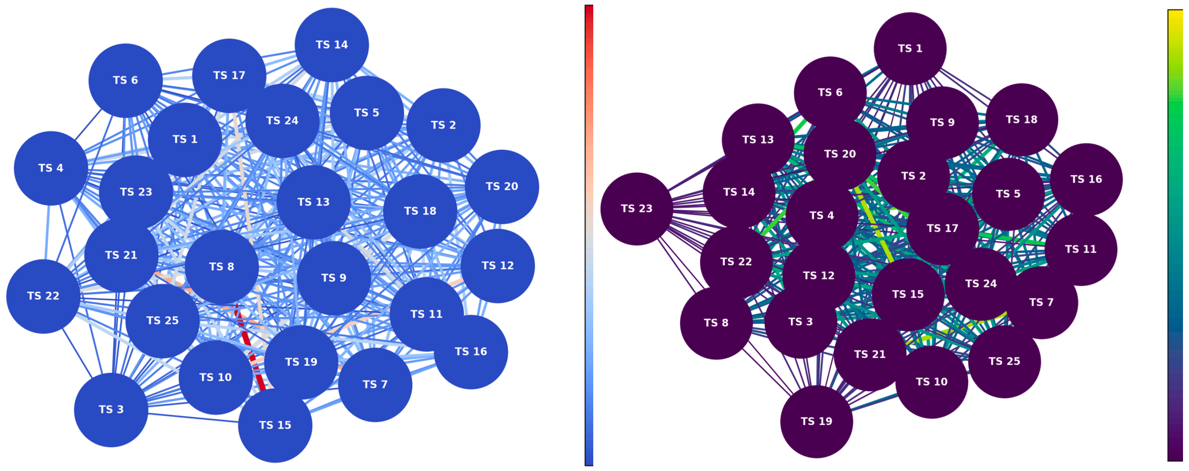

a b

Figure S1. Network of stations analysis of soil similarity matrices for all the parameters: a. wet season b. dry season.

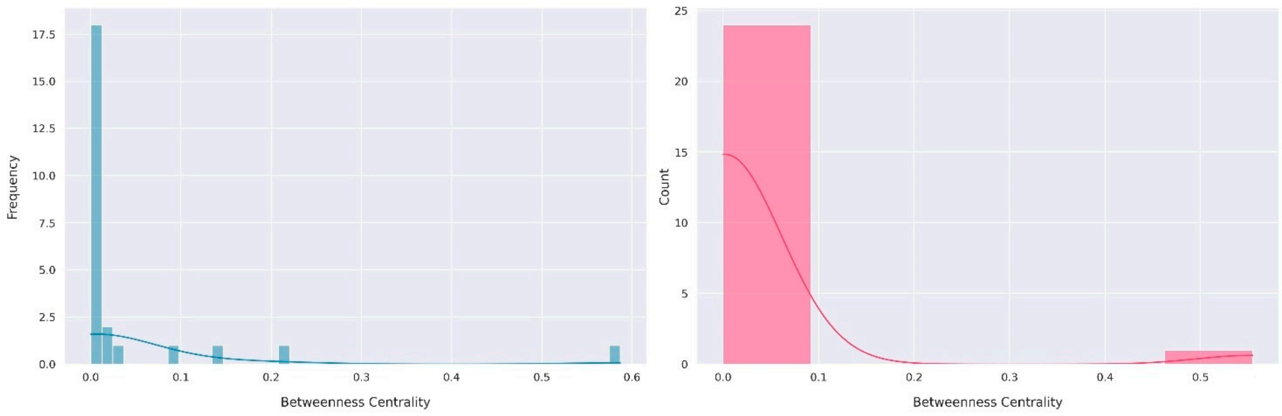

a b

Figure S2. Distribution of betweenness centrality values across the 25 test stations: a. during wet (blue), and b. dry (green) seasons.

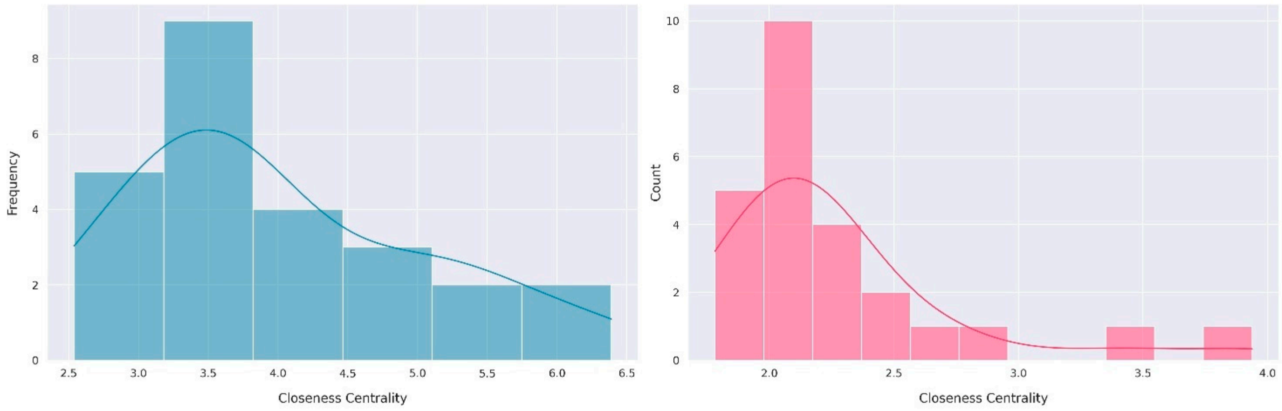

a b

Figure S3. Distribution of closeness centrality values across the 25 test stations: a. during wet and b. dry seasons.

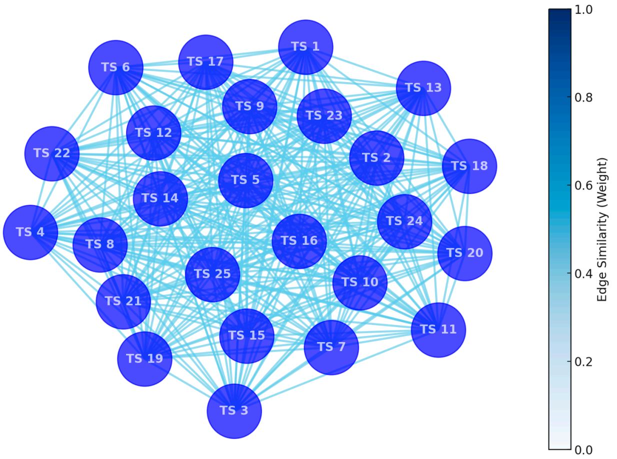

a

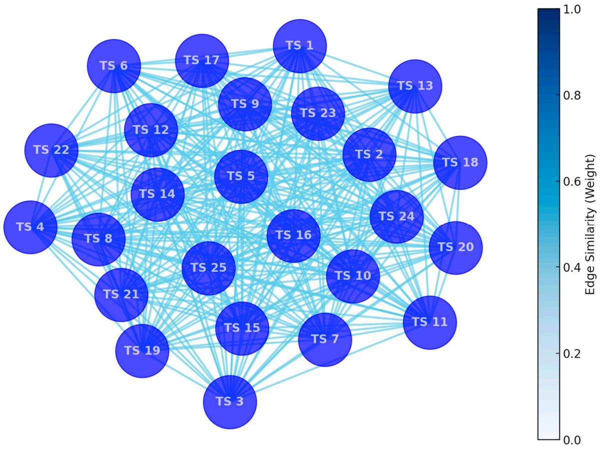

b

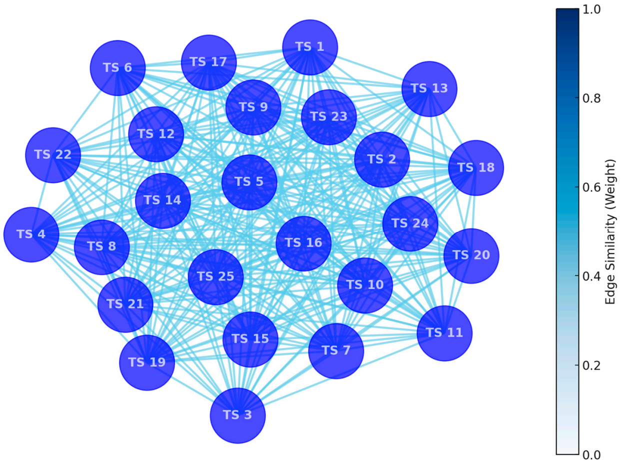

c

Figure S4. Wet season similarity network: a. at a low similarity threshold (θ = 0.05), where the network is fully connected with many weaker relationships between test stations. b. at a moderate similarity threshold (θ = 0.10), where the network maintains significant relationships and filters out weaker connections. c. at a high similarity threshold (θ = 0.15), revealing the most substantial relationships between test stations, with a sparser network structure.

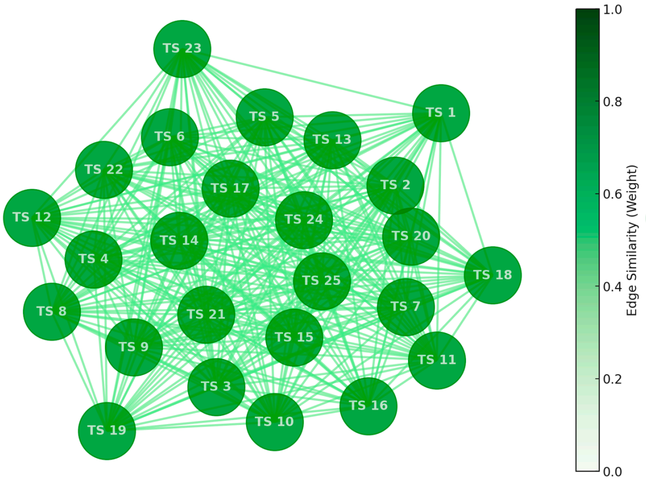

a

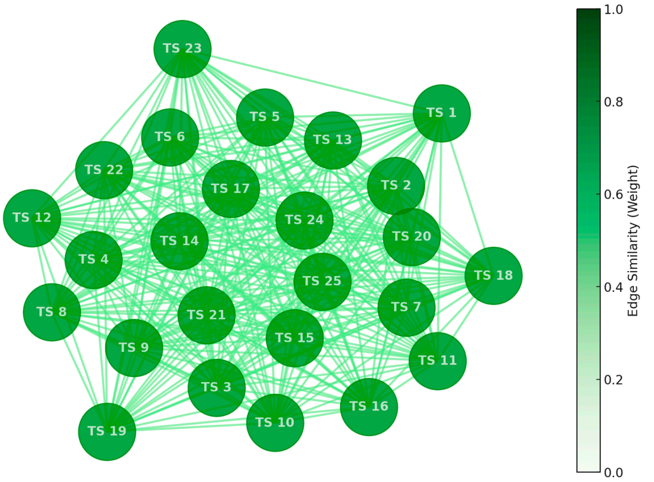

b

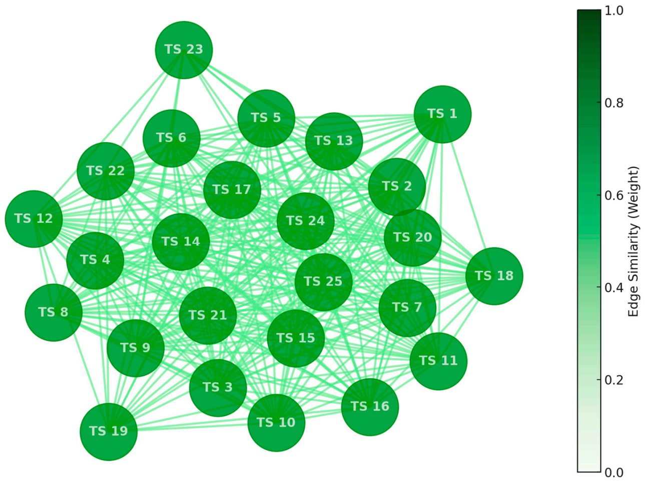

c

Figure S5. Dry season similarity network. a. at a low similarity threshold (θ = 0.05), where the network is fully connected with many weaker relationships between test stations: b. at a moderate similarity threshold (θ = 0.10), where the network maintains significant relationships and filters out weaker connections. c. at a high similarity threshold (θ = 0.15), revealing the most substantial relationships between test stations, with a sparser network structure.

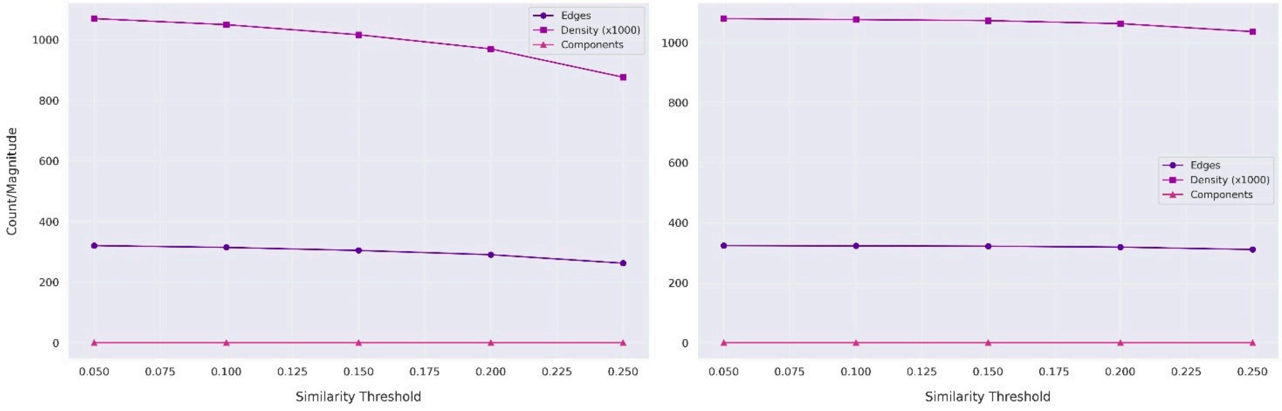

Figure S6. Network distributions of seasons soil properties at different threshold values: a. during wet and b. dry seasons.
